# Key Performance Indicators Used by Dairy Consultants During the Evaluation of Reproductive Performance in a First Visit

**DOI:** 10.3389/fvets.2022.871079

**Published:** 2022-06-23

**Authors:** Ramon Armengol, Lorenzo Fraile, Alex Bach

**Affiliations:** ^1^Department of Animal Science, ETSEA, University of Lleida, Lleida, Spain; ^2^Agrotecnio, University of Lleida, Lleida, Spain; ^3^Marlex Recera i Educació, Barcelona, Spain; ^4^Institució de Recerca i Estudis Avançats (ICREA), Barcelona, Spain

**Keywords:** reproductive performance, first visit, dairy cattle consultant, survey, key parameter

## Abstract

Reproduction plays a fundamental role in the profitability of dairy farms. Consultants use key performance indicators (KPI) to monitor the reproductive performance of the farms. They must decipher between the most suitable ones to face two different scenarios that may need the analysis of different parameters: (1) approach in a first visit, and (2) routine visits. Forty-nine consultants specialized in dairy reproduction from 21 countries responded to an online survey conducted to determine the most suitable parameters in a first visit approach. The survey was comprised of 190 questions, 178 of them rated from 0 (irrelevant) to 10 (maximum importance) points. The questions were divided into 5 sections: (1) consultant and farm model, (2) general data of the farm, (3) cow reproduction, (4) postpartum and metabolic disease, and (5) heifer reproduction. The median, interquartile range, minimum and maximum values, and 95% confidence interval (CI) were determined for each question. Afterward, a multivariate analysis, using between-group linkage *via* Ward's hierarchical clustering was conducted to generate clusters of consultants according to their response pattern. Lastly, a Chi-square test was conducted to assess the association between the years of experience of the consultant and farm size within the clusters generated in each section of the questionnaire. Most of the consultants considered 27 parameters to be highly important to analyze during the first visit. Consultants use several KPIs (in variable quantitative range) to evaluate any of the presented sections. Moreover, consultants preferred parameters focused on heat detection, fertility, and pregnancy achievement regarding the production cycle of a dairy cow. Consultants also showed high interest in obtaining a general overview of milk production, farming efficiency, and the heifer rearing process; but the farm size and the years of experience of the consultant influenced the type and number of parameters chosen as KPI. The parameters rated with the highest importance (rate 10) that could be considered for an easy, fast, and universal first visit to assess the reproductive status were: first service conception rate, overall pregnancy rate, and 21d pregnancy rate for cows, and age at first calving for heifers.

## Introduction

There are multiple biological and management factors that affect reproductive performance in a dairy farm with important repercussions on herd profitability (1). Thus, it is common for dairy producers to seek consultants in setting the best strategy to improve the reproductive performance of the farm. Reproduction consultants in a dairy farm should focus on getting as many cows and heifers pregnant as possible, at the most profitable time of the productive cycle and the lowest cost (2, 3). Moreover, efforts should focus on preventing and controlling multiple factors that can negatively impact reproduction and react as quickly as possible, suggesting actions to correct the identified problems and minimize their consequences.

Advising on reproduction in dairy farms requires considering many aspects beyond those strictly about reproduction (4). Briefly, the most relevant factors include nutrition (5), the farming system and reproductive management (6–8), climate conditions (9), type of farm (10, 11), and periparturient and metabolic and infectious diseases (12–15), among others. Reproduction consultants must then set goals and monitor their accomplishments using key parameters or key performance indicators (KPI) that can be regularly and frequently measured on a farm and should ultimately help to improve the profitability of the herd. These parameters must include factors that may have a positive or a negative impact on the reproductive performance of the farm and should consider biological, as well as economic aspects. These KPIs should quickly respond to changes along time and should be regularly checked by the consultant and (or) the producer.

Typically, advising a dairy farm has two distinct stages: (1) a first visit: where the professional relationship with the farmer begins and a first evaluation of the herd's reproductive performance must be conducted, and (2) continued and routine visits: where the consultant evaluates the changes implemented, suggests improvements, sets goals and identifies and attempts to solve immediate reproductive problems (11). There is a myriad of parameters that can be used as KPI in a dairy farm to assess and monitor reproductive performance. Some of them have been used for many years (i.e., calving interval or age at first calving), while some are new and are progressively being implemented in the farms, like the software and databases, which allow real-time monitoring of parameters, (i.e., days to conception or insemination and pregnancy rates) (16–19). Today's consultants have plenty of information available but, ironically, it may become difficult to select the most suitable KPI to assess reproductive performance on a first visit.

Herein, we aimed that by conducting a survey involving several consultants specialized in dairy reproduction from different countries and production systems we could: 1) assess the most used parameters; and (2) report critical indicators that, according to the consultants surveyed, may improve the effectiveness of the first visit to a dairy farm for reproduction consultancy. Thus, the purpose of this study was to: (1) describe the KPI that consultants specialized in dairy reproduction use to assess the reproductive status of a conventional dairy farm during the first visit; and (2) categorize the different KPIs according to their importance to the consultants; and (3) identify what primary KPI are deemed important by the surveyed consultants and that could be universally used in the first visit to a conventional dairy farm.

## Materials and Methods

### Study Design

This study adopted a descriptive and cross-sectional design based on a survey conducted between January 2019 and May 2021 among consultants of dairy reproduction located in countries in Europe, America, Asia, Africa, and Oceania. The questionnaire was in English and pre-tested by the co-authors and several collaborators. The included parameters and their description were suggested by the authors and reproduction experts, and are commonly found in peer-reviewed articles and dairy veterinarian association reports or publications (i.e., https://www.anembe.com/grupos-de-trabajo/indices-reproductivos/; aabp.org/practitioner- https://journals.tdl.org/bovine/ index.php/bovine/issue/archive-; accessed: July 2018 to December 2018). Moreover, to guarantee the inclusion of the maximum number of KPIs used and to achieve the most accurate and standardized definitions of the parameters, an online search (Pubmed, Scopus, Google Scholar, and Google) was performed using the terms dairy cattle, reproduction and efficiency, management, reproductive performance, pathology, advising, and monitoring.

### Survey and Data Collection

The survey was sent by e-mail to 32 national buiatric associations around the world and directly to 22 consultants. Associations were accessed via google search (accessed: January 2019) using the word combinations: Association & National & Buiatric, Association & National & Veterinary (and then, searching a “dairy cows' section” on the website) or Association & National & Dairy & Veterinarians. When we found a website of one of the associations, we also explored its “interesting links section” and accessed websites of other associations that could be linked. Direct contact with consultants was made *via* professional email. In addition, all the receivers of the survey were encouraged to re-send the survey to other consultants. The survey did not entail any remuneration, prize, or draw for participants. The participants responded to the questionnaire anonymously. The mailed survey included a cover letter detailing the objectives of this work and the conditions of participation (placing them on a first visit scenario and assuming that the farm being visited could provide all the parameters included in the survey), along with a link to an online Google Forms questionnaire. A reminder card was sent 2-months after each mailing. A power analysis using public epidemiologic software (https://epitools.ausvet.com.au/) was conducted before sending the survey and indicated that 43 answers would be necessary to correctly estimate the mean for each question with a confidence level of 95% and a potency of 80%, assuming a standard deviation (SD) of 4 and an accepted absolute error of 1.2.

### Questionnaire Sections

The survey was comprised of 190 questions divided into five sections. Each question included a clear definition of each parameter and its calculating equation (if applicable). The complete survey is available as Supplementary Material (Supplementary Table 1 in Supplementary Material).

#### Section 1: Consultant and Farm Model

This first section included 12 questions that assessed baseline descriptive information about the responding consultants and their farms. Questions related to personal information included age, years of experience, country; and common farm characteristics inquired about herd size, housing types, breed, and several milkings per day. Depending on the question, the participant had to write an answer or choose between different ranges or options.

#### Section 2: General Data of the Farm

Questions in this section included 43 candidate parameters that potentially could become a KPI. These parameters provide information about the distribution of the cows in the herd (i.e., milking cows and dry cows), the age and physiological status of the cows (i.e., number of lactation and average days in milk (DIM), and the number of pregnant cows), milk yield, and incidence of infectious diseases.

#### Section 3: Cow Reproduction

Questions in this section comprised 49 parameters potentially useful to evaluate the reproductive performance of cows in a dairy herd. The parameters proposed as potential KPIs in this section provided information on fertility, heat detection, time at pregnancy, and pregnancy loss, among others. This section also included parameters that monitor the probability of pregnancy and the time at which it occurs, such as the first service conception rate (CR) (The term “conception rate” is used to indicate “conception risk” throughout the manuscript), overall pregnancy rate, or voluntary waiting period. Thus, some of the KPIs included could be useful in assessing the efficiency of the farm's reproductive strategy.

#### Section 4: Postpartum and Metabolic Disease

This section consisted of 36 parameters related to the postpartum period and its associated afflictions. The parameters provided information about the type of calving and incidence of uterine and metabolic diseases that may hurt the future reproductive performance of cows (i.e., % metritis and % retained placenta, % hypocalcemia, or % Ketosis).

#### Section 5: Heifer Reproduction

This final section included 50 parameters that could potentially be used to evaluate heifer reproductive performance and their rearing process. The KPI candidates in this section provided information on fertility, heat detection, time at pregnancy, pregnancy loss, age, and growth of heifers, among others. This section also included parameters that can be useful as a KPI, not only for reproduction but also for assessing the efficiency of the entire rearing process. For example, age at first calving or percentage of heifers calving of <24 months old.

Participants were asked to indicate the relevance or usefulness of each parameter in sections 2, 3, 4, and 5 using an eleven-point scale: from 0 (not important at all) to 10 (highly important). To ease the interpretation of the results obtained, answers were grouped into four categories: Highly relevant (score ≥8), moderately relevant (score between ≥5 and <8), low interest (score between ≥2 and <5), and irrelevant (score <2).

### Statistical Analyses

A descriptive statistic was initially carried out. Thus, the median, interquartile range, minimum and maximum values (min-max), and confidence interval (CI) of 95% were determined for each question of the questionnaire. Afterward, a multivariate analysis, using between-group linkage *via* Ward's hierarchical clustering was conducted to generate clusters of consultants according to their response pattern, considering the answers from sections 2, 3, 4, and 5 of the questionnaires, and representing them in a constellation plot. This multivariate technique allows obtaining a pattern of response for all the questions without assuming any distribution of the data. The number of clusters was set up by obtaining the best goodness of fit with this multivariate analysis technique. Years of experience and farm size were discretized and analyzed as ordinal variables. Thus, years of experience included the groups: <15, 15–20, 21–25, and >25 years, and the size of the farm included the groups: 1–150, 151–300, 301–700, and >700 cows. Lastly, it was considered interesting enough to decipher any potential association between years of experience and the size of the farm with the pattern of response to the questions. Thus, a chi-square test was conducted to assess the association between the years of experience of the consultant and farm size within the clusters generated in each section of the questionnaire.

## Results and Discussion

Forty-nine consultants from 21 countries answered the questionnaire. The number of responders was lower than expected, averaging only 2.3 consultants per country (range, 1 to 7), and many countries were not represented, which may pose a significant bias in the data presented. Nevertheless, as discussed later, responders included consultants from several countries at different stages of their careers. The results of the survey were split into 5 sections, which are presented below. Two important matters were clearly explained in the cover letter for participants: It was assumed the respondent was evaluating a dairy farm on the first visit, and that the consultant could obtain information about all the parameters included in the survey. None of the respondents commented on the impossibility of obtaining any of the parameters included in the suggestions/comments section of the survey.

### Section 1: Consultant and Farm Model

The questionnaires were answered by the consultants from 21 countries as follows: Belgium (2), Brazil (1), Canada (3), Chile (1), Colombia (1), Denmark (3), Estonia (1), France (3), Germany (2), Greece (2), Israel (2), Italy (2), Netherlands (2), Peru (1), Poland (1), Portugal (3), Spain (7), Sweden (4), United Kingdom (1), Uruguay (1), and United States of America (6).

The age of the consultants ranged between 31 and 70 years (16 between 31 and 40 years old; 17 between 41 and 50; 8 between 51 and 60; 8 between 61 and 70) and reporting <15 years of experience (14; 28.57%), between 15 and 20 years (13; 26.53%), between 20 and 25 years (9; 18.37%), and >25 years (13; 26.53%).

Regarding the farming system, 36 out of 49 consultants surveyed (73.4%) stated that the most common farming system in their practice was a closed farm where cows had no access to pasture. A small set of consultants (13; 26.5%) stated that the most common farming system in their practice consisted of cows with access to pasture at any time of the productive cycle. The questionnaire allowed us to define three types of housing systems: Tie-stalls (0;0.0%), free-stalls or cubicles (40; 81.6%), and bedded pack or loose-housed (9; 18.4%). Farm size distribution, which referred only to lactating cows, was 1 to 150 (12; 24.49%), 151–300 (12; 24.49%), 301-700 (13; 26.53%), >700 (12; 24.49%), and most of the consultants worked with farms milking twice daily (33; 67.3%). Farms with a devoted close-up pen were referenced in 34 of the returned questionnaires (69.4%).

Most consultants (35; 71.4%) worked with Holstein Friesian (HF), with the remainder working with HF and other breeds (13; 26.5%), such as Swedish Red (4), Jersey (5), Montbéliard (2), Brown Swiss (1), and their crosses (2). Jersey was selected as the main dairy breed by one consultant. The main breeding technique was artificial insemination (AI) (47; 95.9%), whereas natural mating and embryo transfer were reported once each (2%); with year-round calving (46; 93.8%). Seasonal calving was reported as a production model in only 3 of the returned questionnaires (6.1%).

The participants were also asked about the ideal period of the data they would request to assess reproduction in a first visit. Ten out of 49 consultants (20.4%) would analyze the data of the current year, 9 (18.4%) from the last year, 1 from the last 6 months (2.0%), 15 (30.6%) from the last 3 years, 10 from the last 5 years (20.4%), and 4 from the last 10 years (8.1%). There exist two ways of looking at data: 1) to describe the evolution of a given parameter over time, and 2) to decide about a particular aspect of the farm. If the aim is to draw the evolution of a given parameter, enrolling historical data may be useful. However, for a first visit, if the intention is to identify areas of potential improvement and decide where to act, obtaining recent historic data is important but collecting historic data from past years may have limited value depending on the goal. In the current survey, ~80% of consultants would gather data from the previous year and before without considering that the relevance of a first visit approach could be low.

### Section 2: General Data of the Farm

Out of the 43 parameters that could be evaluated in this section, participants selected eight as highly important−1st lactation cows (%), pregnant cows (%), average DIM (n), days dry (n), 305 DIM yields (kg), culling rate (%), cows culled for a reproductive reason (%), and herd status for bovine viral diarrhea virus (BVDV) (present/absent); 30 as moderately important, 4 were of low interest, and one was irrelevant (see Table 1 for detailed data).

**Table 1:** Answers given by consultants about farm general parameters, where 0 is of minimal or irrelevant importance and 10 represents the maximum importance.

**General data of the farm**
**parameter**	**Median**	**CI 95%**	**Interquartile range**	**Min–max**
Culling rate, %	8	6.86–8.36	3.0	0–10
Average days dry, d	8	5.80–7.34	3.5	0–10
Pregnant cows, %	8	5.88–7.53	3.5	0–10
305 days yield, kg	8	6.02–7.68	3.5	0–10
Cows culled for reproductive reasons, %	8	6.41–8.03	3.5	0–10
Herd status for BVDV, present/absent	8	5.37–7.40	4.0	0–10
1^st^ lact cows, %	8	5.78–7.60	4.0	0–10
Average DIM, d	8	6.13–7.82	4.0	0–10
Average SCC, SCC/mL	7	5.48–7.04	2.0	0–10
“Do Not Breed” cows, %	7	6.01–7.45	2.0	0–10
Peak milk 3^rd^ lact cows, kg	7	5.18–6.81	3.0	0–10
Peak milk 1^st^ lact cows, kg	7	5.27–6.96	3.0	0–10
Replacement, %	7	6.14–7.73	3.0	0–10
Lameness, %	7	4.69–6.35	3.5	0–10
Number of milking cows, *n*	7	5.85–7.41	4.0	0–10
Lactating cows daily milk yield, kg	7	5.54–7.14	4.5	0–10
Total number of cows, *n*	7	6.23–7.72	4.5	0–10
Failure to conceive culling rate, %	7	4.92–6.98	5.0	0–10
Average number of lactation, *n*	7	6.38–7.85	5.0	2–10
Average DIM culled cows, *n*	7	5.01–6.98	4.0	0–10
Average lact number of culled cows, *n*	7	5.03–6.79	4.0	0–10
Herd status for neosporosis, present/absent	7	4.38–6.38	6.0	0–10
Cows culled for lameness reason, %	6	4.23–5.97	3.5	0–10
Cows culled for mastitis reason, %	6	4.36–6.04	3.5	0–10
Clinical mastitis, %	6	4.59–6.30	3.5	0–10
Number of 1^st^ lact cows, *n*	6	5.23–6.88	3.5	0–10
Dry cows, %	6	5.04–6.87	3.5	0–10
Monthly milk yield, kg	6	4.54–6.39	4.5	0–10
Peak milk 3^nd^ lact cows, DIM	6	4.27–6.09	5.0	0–10
Peak milk 1^st^ lact cows, DIM	6	4.56–6.41	5.0	0–10
Herd status for IBR–IPV, present/absent	6	4.22–6.34	7.5	0–10
All cows daily milk yield, kg	5	4.09–6.07	3.0	0–10
Number of dry cows, *n*	5	3.89–5.65	3.5	0–10
Daily milk yield, kg	5	4.00–5.66	5.0	0–10
Peak milk 2^nd^ lact cows, kg	5	4.23–6.16	5.0	0–10
Peak milk >3^rd^ lact cows, DIM	5	2.99–5.00	7.0	0–10
Peak milk >3^rd^ lact cows, kg	5	3.52–5.53	7.5	0–10
Peak milk 2^nd^ lact cows, DIM	5	3.56–5.62	8.0	0–10
Cows culled for accident reason, %	4	3.00–4.75	5.5	0–10
Total number of pregnant cows, *n*	4	2.72–4.58	6.0	0–10
Number of cows culled, *n*	3	2.66–4.31	6.0	0–10
Herd status for brucellosis, present/absent	2	2.72–5.11	8.5	0–10
Herd status for FMD, present/absent	0	1.64–3.74	6.5	0–10

*Results are expressed as median, CI 95%, interquartile range and minimum (Min), and maximum (Max) values. Parameters are sorted according to the median obtained, from highest to lowest*.

*CI, Confidence interval 95%; DIM, Days in milk; lact, lactation; SCC, Somatic cell count; BVDV, Bovine Viral Diarrhea Virus; IBR–IPV, Infectious Bovine Rhinotracheitis – Infectious Pustular Vulvovaginitis; FMD, Foot and Mouth Disease*.

The parameters classified as highly important in this section might give a general view of the farm efficiency and profitability of the cows since the viability of a dairy farm is highly dependent on milk production and reproductive performance (20). However, these parameters bear a considerable lag, and, thus, may not help to detect a current problem. For instance, the percentage of pregnant cows reflects the reproductive performance of many months ago and if the farm had a conception problem; this indicator would take quite a long time to change, making it unsuitable to make decisions. The inclusion of health status regarding BVDV is noteworthy as a pathogen of most concern to the consultants; however, considering specific pathogens as a “highly important KPI” depends on the region or country where the farm is located. Thus, the inclusion of BVDV as a very important KPI can be explained by the fact that most countries represented in this study have confirmation of the presence of this virus in their bovine population, which may cause consultants to want to control this virus as much as possible due to its negative impact on reproductive performance (21).

Nevertheless, the maximum median of the parameter importance in this section was 8, at the lowest value within the category of highly important KPI (9–11), reinforcing the notion that these indicators may not be critical to assessing reproductive performance on the first visit in a dairy farm. The highlighted parameters in this section may be useful in finding out if in the past there were problems affecting reproductive performance, but nothing more. As an example, to have a certain current view of reproductive performance or even to make a certain forecast, the consultant should get insights into the distribution of the DIM that provides the average DIM, or what is the distribution according to the days of pregnancy in cows, from which the % of pregnant cows is obtained. Regarding culling rate and culling reasons, as much as consultants and scientists insist on classifying the reasons for culling, in practice, it becomes an almost impossible task as culling decisions are seldom based on a single parameter [i.e., % of cows culled for a reproductive reason, valued 8 (6.41–8.03) in Table 1], and typically results from a combination of causes, such as genetic merit, level of production, reproduction, disease, or stocking density. Two parameters that consultants could also check include the expected number of calvings and number of dry cows for an upcoming period, which can give an overview of the distribution of current pregnancies, according to the DIM and days of pregnancy. These 2 parameters (expected number of calvings and number of dry cows) were not included in the candidate KPI parameters and, thus, cannot be assessed herein. However, the consultant should bear in mind, that these data are estimations, and may not be accurate as there will likely be some pregnancy losses.

Responders were grouped into five clusters for section 2 (Supplementary Figure 1 in Supplementary Material). Cluster 1 included the highest number of consultants (24; 49.0%), where most of the questions were considered moderately or highly important, except for total pregnant cows, percentage of cows culled for accidental reasons, and herd status for Foot and Mouth Disease (FMD). On the other hand, cluster 3 included 24.5% (*n* = 12) of the consultants that considered 3, 17, 18, and 5 questions irrelevant, low important, moderately important, and highly important, respectively. Cluster 2 included 12.2% (*n* = 6) of the consultants that considered 12, 11, 15, and 5 questions irrelevant, low importance, moderate importance, and high importance, respectively. Lastly, clusters 4 and 5 included the lowest number of consultants (4; 8.1 and 3; 6.1%, respectively). In cluster 4, most of the questions were considered irrelevant or of low importance except for an average number of lactations, percentage of 1st lactation cows, percentage of cows culled due to lameness, and herd status for BVDV that were rated as moderately or highly important. Cluster 5 included 6.1% of the consultants (*n* = 3), where 8 parameters were considered highly important (total number of cows, number of milking cows, percentage of 1^st^ lactation cows, monthly milk yield, daily milk yield, lactating cows daily milk yield, all cows daily milk yield, and failure to conceive culling rate), 7 as moderately important, and the rest were considered of low importance or irrelevant (for detailed data see Supplementary Table 2 in Supplementary Material).

The parameters considered highly important for, at least, 50% of the consultants were: culling rate for 85.7% of the consultants (*n* = 42); pregnant cows (%), average DIM, and proportion of cows culled for the reproductive reason for 73.5% of the consultants (*n* = 36), 305 yields (kg) for 61.2% of the consultants (*n* = 30), percentage of 1^st^ lactation cows, and herd status for BVDV for 57.1% of the consultants (*n* = 28) (see Table 2 for detailed data). Lastly, the length of the dry period was considered highly important by 48.9% of the consultants surveyed (*n* = 24). The length of the dry period is usually well-defined and controlled by the farmer and has a standard length of ~ 60 d. However, the length of dry period may be unduly prolonged if, due to reproductive problems, pregnancies are achieved too late. The farmer is then forced to dry the cows by economic decisions dictated by a low milk yield rather than based on days of pregnancy. This could be the reason why almost 50% of the consultants surveyed considered it important to review the length of the dry period on a first visit to the farm.

**Table 2:** Parameters evaluated as highly important (range 8–10) on Section 2: General Data of the Farm used by, at least, 50% of the consultants surveyed after hierarchical clustering analysis.

**General data of the farm**	**Median**	**CI 95%**	**Interquartile**	**Min–max**	**% of consultants rating**	**Clusters mainly rating the**
			**range**		**as highly important**	**parameter as highly important**
Culling rate, %	8	6.86–8.36	3.0	0–10	85.7 (42)	1, 2, 3
Pregnant cows, %	8	5.88–7.53	3.5	0–10	73.5 (36)	1, 3
Average DIM, d	8	6.13–7.82	4.0	0–10	73.5 (36)	1, 3
Cows culled for reproductive reason, %	8	6.41–8.03	3.5	0–10	73.5 (36)	1, 3
305 d yield, kg	8	6.02–7.68	3.5	0–10	61.2 (30)	1, 2
Herd status for BVDV, absent/present	8	5.37–7.40	4.0	0–10	57.1 (28)	1, 4
1^st^ lact cows, %	8	5.78–7.60	4.0	0–10	57.1 (28)	1, 4

*Results are expressed in median, CI 95%, interquartile range, minimum (Min) and maximum (Max) values, and proportion of consultants that rated the parameter as highly important (n in brackets). The clusters obtained after hierarchical clustering analysis where most of these highly important parameters were present are also depicted. Parameters are sorted according to proportion of consultants that rated the parameter as highly important, from highest to lowest*.

*CI, Confidence interval 95%; DIM, Days in milk; lact, lactation; BVDV, Bovine Viral Diarrhea Virus*.

It is interesting to observe whether the characteristics of the consultant reflected in Section 1 (years of experience) or the farm size, can be associated with the described clusters of consultants in the other sections that composed the survey. In this section, the years of experience were not associated with the membership to a particular cluster whereas farm size was significantly associated with the described clusters (*P* = 0.014). Thus, consultants that advise farms between 301 and 700 milking cows and farms with >700 milking cows are mostly associated with cluster 1 and clusters 4 and 5, respectively (see Supplementary Figure 2 in Supplementary Material). This association implies that consultants of farms between 301 and 700 milking cows consider it important to evaluate a larger number of general data on the production and reproduction, such as peaks of milk, culling rate, percentage of “do not breed” cows, average DIM, the proportion of cows culled for reproductive reasons, and incidence of infectious diseases; compared with consultants advising farms with >700 dairy cows, who focused on a lower number of parameters mainly referring to census and production, such as a number of cows or milk yield. With these results, it seems that consultants of farms milking between 301 and 700 cows are more likely to consider KPIs that primarily reflect the past. On the other hand, farm consultants with >700 cows tend to consider KPIs that reflect the present.

### Section 3: Cows' Reproduction

This section included 49 parameters, 17 were evaluated as highly important, 28 were considered as moderately important, four were rated as low important and any of them was considered as irrelevant considering the median value for each parameter. Of the parameters considered highly important, three obtained a maximum rating of 10 (overall pregnancy rate, first service CR, and 21d. pregnancy rate), and anotherthree obtained a rating of nine (voluntary waiting period, CR, and heat detection rate), and the rest were rated at 8 (percent of conceived cows over total cows served, days open, percentage abortion >90 days, CR synchronized cows, CR first service in 1st lactation cows, CR first service in multiparous cows, CR 1^st^ lactation cows, CR multiparous cows, services per pregnancy, calving to first service interval, and percentage of cows not pregnant >200 DIM) (see Table 3 for detailed data).

**Table 3:** Answers given by consultants on Section 3: Cows' Reproduction data parameters, where 0 is of minimal or irrelevant importance and 10 represents the maximum importance.

**Cows' reproduction**
**parameter**	**Median**	**CI 95%**	**Interquartile range**	**Min–max**
First service CR, %	10.0	7.74–9.23	2.0	0–10
Overall pregnancy rate, %	10.0	6.97–8.82	3.0	0–10
21d. Pregnancy rate, %	10.0	6.10–8.30	5.5	0–10
Heat detection rate, %	9.0	6.89–y8.57	3.0	0–10
CR, %	9.0	7.43–8.93	3.0	0–10
Voluntary waiting period, d	9.0	7.49–8.75	3.0	1–10
Abortion >90 days, %	8.0	6.31–7.85	3.0	0–10
Days open, *n*	8.0	6.20–8.08	4.0	0–10
CR first service in 1^st^ lact cows, %	8.0	6.45–8.24	4.0	0–10
Conceiving of served, %	8.0	5.57–7.56	4.5	0–10
Calving to first service interval, d	8.0	6.31–8.09	4.5	0–10
CR multiparous cows, %	8.0	5.76–7.71	5.0	0–10
CR first service in multiparous cows, %	8.0	5.80–7.86	5.0	0–10
CR 1^st^ lact cows, %	8.0	5.93–7.86	5.0	0–10
CR synchronized cows, %	8.0	5.75–7.79	5.5	0–10
Services per pregnancy, *n*	8.0	5.49–7.52	7.0	0–10
Cows not pregnant >200 DIM, %	8.0	4.68–6.86	8.0	0–10
Calvings per month, *n*	7.0	5.07–6.71	3.0	0–10
Pregnancy loss (1–90days), %	7.0	5.29–6.99	3.0	0–10
CR of inseminators, %	7.0	5.10–6.81	3.5	0–10
Days at pregnancy diagnosis, n	7.0	5.40–7.16	3.5	0–10
Calving interval, d	7.0	5.39–7.33	4.0	0–10
Interval service to service, d	7.0	5.81–7.44	4.0	0–10
18–24 d. return to service/heat, %	7.0	5.12–7.03	5.0	0–10
Pregnancy loss, %	7.0	6.05–7.81	5.0	0–10
CR first Service in 3^rd^ lact cows, %	7.0	4.53–6.65	6.5	0–10
CR first Service in 2^nd^ lact cows, %	7.0	4.98–7.09	6.5	0–10
Herd calving to conception interval, d	7.0	4.90–7.17	9.0	0–10
1^st^ lact cows calved, %	6.0	4.27–5.92	4.5	0–10
Cows not pregnant >150 DIM, %	6.0	4.49–6.56	6.5	0–10
Interval heat to heat, d	6.0	4.75–6.63	5.0	0–10
CR first service in >3^rd^ lact cows, %	6.0	4.22–6.29	6.5	0–10
CR 2^nd^ lact cows, %	6.0	4.16–6.20	8.0	0–10
2–17 d. return to service/heat, %	5.0	3.91–5.79	6.0	0–10
36–48 d. return to service/heat, %	5.0	3.98–5.97	6.0	0–10
25–35 d. return to service/heat, %	5.0	4.29–6.27	6.0	0–10
Submission rate first 3 weeks, %	5.0	3.97–6.02	6.5	0–10
CR of the sire, %	5.0	3.28–5.32	7.0	0–10
Cows served <90 DIM, %	5.0	3.29–5.32	7.0	0–10
Anovulatory cows, %	5.0	3.58–5.63	7.0	0–10
>49 d. return to service/heat, %	5.0	3.81–5.85	7.0	0–10
Early pregnancy loss (1–42 days), %	5.0	3.47–5.58	7.5	0–10
Submission rate, %	5.0	3.36–5.36	8.0	0–10
CR >3^rd^ lact cows, %	5.0	3.49–5.52	8.0	0–10
CR 3^rd^ lact cows, %	5.0	3.82–5.80	8.0	0–10
100-day In-calf rate, %	4.0	2.81–4.77	7.0	0–10
Days to culling, *n*	3.0	2.32–4.08	6.0	0–10
Non-return rate, %	3.0	2.56–4.45	7.0	0–10
Ovarian cysts, %	3.0	2.91–5.00	7.5	0–10

*Results are expressed as median, CI 95%, interquartile range and minimum (Min) and maximum (Max) values. Parameters are sorted according to the median obtained, from highest to lowest*.

*CI, Confidence interval 95%; CR, Conception rate; DIM, Days in milk; lact, lactation*.

It is noteworthy that in this section none of the parameters was considered irrelevant and that 17 parameters were considered critical to evaluate the reproductive performance of a farm during a first visit. Essentially, the parameters evaluated with the greatest marks (8–10) are probably sufficient to provide an accurate view of the reproductive status of a farm and its efficiency in the past. These parameters bear considerable lag, since some of them need a full cow cycling period (~21 days) to be obtained (i.e., heat detection rate), or even more days to reach a pregnancy diagnosis (roughly 28–35 d post-breeding) (i.e., conception and pregnancy rate. The parameters that monitor pregnancy loss or time when cows achieve pregnancy at the most optimal time requires a longer time to be obtained. If these parameters are not optimal in a farm, clearly, the problem has already occurred, and that the consultant will not be able to solve it (or it may even already be solved). However, it is positive and important for the consultants to consider them in their first visit, as it may help to diagnose historical problems in the reproductive performance of the farm and the consultants should take measures to ensure that these problems do not occur in the future regardless of their occurrence in the past. In this regard, the consultants interviewed have chosen the parameters that monitor the costliest pregnancy losses (> 90 d pregnancy loss) and the most economically critical time for a cow to remain open (> 200 DIM) (22).

The fact that 31 of the parameters have been scored as moderately important may suggest two aspects: either some consultants do not differentiate between a first approach and routine reproduction visits of a farm, or some consultants involuntary tend to collect more information than necessary. As an example, considering highly important parameters that indirectly show past CRs (i.e., number of services per pregnancy) or that aim at evaluating the efficiency of achieving the cows' pregnancy at the right time through averages (i.e., days open) can lead to a considerable bias for different reasons: they may fluctuate greatly during the requested period or because the cows included have changed status (i.e., “eligible to be bred” *vs*. “do not breed” cow). Even more important, focusing on days open to assess reproduction is extremely dangerous. Since days open are defined as the number of days between calving and a breeding date, resulting in a confirmed pregnancy, they can only be calculated once pregnancy is confirmed. Some computer software, though, may consider that the cow becomes pregnant at the day that days open are calculated, but of course, that is providing a false representation of the reproductive status of the herd. An extreme example of the danger of days open could be found in a situation where an entire herd could be composed of open cows with reproductive problems except one single cow that became pregnant at 88 DIM; and days open would be very optimal: 88 d, unless days open were calculated considering that all non-pregnant cows conceived at the day of calculation, which would not be a valid representation of reality either.

Consultants were grouped into five clusters through the hierarchical analysis for section 3 (see Supplementary Figure 3 in Supplementary Material). Clusters 1, 2, and 3 accounted for 87.7% of the respondents (*n* = 43). There was a major cluster (1) that grouped 55.1% of the consultants (*n* = 27) and most of the parameters were considered as highly (23) or moderately (24) important except percentage of ovarian cysts, submission rate, 2–17 d percentage return to service/heat, 100-Day In-calf rate and cows served <90 DIM than were rated as of low importance. Clusters 2 included 18.4% of the consultants (*n* = 9) and rated 13, 14, 15, and 8 of the parameters as highly, moderately, low importance, and irrelevant, respectively. Cluster 3 grouped 14.3% of the surveys answered (*n* = 7), where three parameters were considered as highly important, 20 as moderately important, and 27 as of low importance. Finally, clusters 4 and 5 included the lowest proportion of consultants (3; 6.1% in each cluster). In cluster 4, most of the parameters were classified as highly important (25) or irrelevant (15). Cluster 5, although representing only 6.1% of the consultants, surprisingly rated almost all the parameters as irrelevant [43] and the rest as low important (7). In general, parameters that monitor pregnancy rates and CRs were the top-rated. Of special interest were the first service pregnancy rate and CRs divided between 1st lactation and multiparous cows, rather than specific conception rates for each lactation (1st, 2nd, 3rd, or >3rd lactations) (see Supplementary Table 3 in Supplementary Material for detailed data). It is logical that on a first visit, the consultants do not consider it a priority to independently analyze CRs for each lactation. This analysis would be more recommended during routine and continuous visits, maximizing accuracy in the identification of particular CR problems in certain groups of cows. A separate analysis of primiparous and multiparous CRs is important on a first visit, as differences in reproductive management and the physiology between 1st lactation and multiparous cows are substantial (22–24).

Considering the highest averages obtained from the evaluated parameters, the first service CR was rated highly important by 93.8% of the respondents (*n* = 46); the overall pregnancy rate by 87.7% (*n* = 43); the voluntary waiting period and the CR by 79.6% (*n* = 39); heat detection by 75.5% (*n* = 37); 21d. Pregnancy rate, first service and overall CR for both primiparous and multiparous cows, days open and percentage of cows open at > 200 DIM by 73.5% (*n* = 36); Percent conceiving of served, CR of synchronized cows, and calving to first service interval by 61.2% (*n* = 30). Lastly, services for pregnancy and the percentage of abortions at >90 days were consied as highly important by 18.4% (*n* = 9) and 6.12% (*n* = 3) of respondents, respectively (see Table 4 for detailed data).

**Table 4:** Parameters evaluated as highly important (range 8–10) on Section 3: Cows' Reproduction used by, at least, 50% of the consultants surveyed after hierarchical clustering analysis.

**Cows' reproduction**	**Median**	**CI 95%**	**Interquartile**	**Min–max**	**% of consultants that rated**	**Clusters mainly rating the**
**parameter**			**range**		**the parameter as**	**the parameter as**
					**highly important**	**highly important**
First service CR, %	10.0	7.74–9.23	2.0	0–10	93.8 (46)	1, 2, 3, 4
Overall pregnancy rate, %	10.0	6.97–8.82	3.0	0–10	87.7 (43)	1, 2, 3
Voluntary waiting period, d	9.0	7.49–8.75	3.0	0–10	79.6 (39)	1, 2, 4
CR, %	9.0	7.43–8.93	3.0	0–10	79.6 (39)	1, 2, 4
Heat detection rate, %	9.0	6.89–8.57	3.0	0–10	75.5 (37)	1, 3, 4
Days open, d	8.0	6.20–8.08	4.0	0–10	73.5 (36)	1, 2
Cows not pregnant >200 DIM, %	8.0	4.68–6.86	8.0	0–10	73.5 (36)	1, 2
21d. Pregnancy rate, %	10.0	6.10–8.30	5.5	0–10	73.5 (36)	1, 2
CR first service in 1^st^ lact cows, %	8.0	6.45–8.24	4.0	0–10	73.5 (36)	1, 2
CR first service in multiparous cows, %	8.0	5.80–7.86	5.0	0–10	73.5 (36)	1, 2
CR 1^st^ lact cows, %	8.0	5.93–7.86	5.0	0–10	73.5 (36)	1, 2
CR multiparous cows, %	8.0	5.76–7.71	5.0	0–10	73.5 (36)	1, 2
Calving to first service interval, d	8.0	6.31–8.09	4.5	0–10	61.2 (30)	1, 4
CR synchronized cows, %	8.0	5.75–7.79	5.5	0–10	61.2 (30)	1, 4
Percent conceiving of served, %	8.0	5.57–7.56	4.5	0–10	61.2 (30)	1, 4

*Cows' Reproduction used by, at least, 50% of the consultants surveyed after hierarchical clustering analysis*.

*Results are expressed as median, CI 95%, interquartile range, minimum (Min) and maximum (Max) values, and proportion of consultants that rated the parameter as highly important (number of observations within brackets). The clusters obtained after hierarchical clustering analysis, where most of these highly important parameters were present are also depicted. Parameters are sorted according to the proportion of consultants that rated the parameter as highly important, from highest to lowest*.

*CI, Confidence interval, 95%; CR, Conception rate; DIM, Days in milk; lact, lactation*.

The probability of getting a cow pregnant depends on the combination of the probability of insemination (heat detection, hormonal synchronization, or both) and CR (3). Therefore, heat detection, fixed-time insemination, or both may be essential for most consultants (26–28). Not only it is important to get the cow pregnant but to do it as efficiently as possible. This means doing it at the lowest cost (i.e., hormones, labor, and technology) but also at the desired time (first service) of the cow's productive cycle depending on age, number of cow lactation, farm costs, and income (2). In this sense, the inclusion of first service CR (%) and overall pregnancy rate (%) as the top KPI is logical since consultants are evaluating the reproductive performance of a farm during a first visit.

Parameters that were not included in highly important KPI by more than 50% of consultants are parameters that could be useful in a deeper analysis and, over time, to identify specific problems that do prevent cows from reaching pregnancy at the right time or an insufficient rate. They could even identify if the reproductive problem is in a particular group of cows, bulls, or technicians. This kind of evaluation does not have any primary interest in a first visit. According to the experts interviewed, the use of a maximum of 15 KPIs should be sufficient to assess the reproductive performance of a farm on a first visit. These parameters focus on heat detection, conception rate, and when the pregnancy is achieved.

The distribution of consultants in the five clusters of section 3 was also affected by the size of the farm (*P* = 0.019), but not by the years of experience of the consultant (*P* >0.05). Thus, 84.6% of consultants advising farms between 301 and 700 milking cows are concentrated in cluster 1, whereas clusters 4 and 5 are mostly composed (83.3%) of veterinarians who work on farms with >700 milking cows (see Supplementary Figure 4 in Supplementary Material). Both groups of consultants consider almost the same number of parameters as highly important (22 parameters in cluster 1 and 23 parameters in clusters 4 and 5 altogether). Of special interest is that consultants who advise farms with more than 700 milking cows differentiate the parameters as highly important or irrelevant, rating very few parameters as low or moderately important, compared to the consultants who are advising farms with 301–700 milking cows, where none of the parameters was rated as irrelevant.

### Section 4: Postpartum and Metabolic Diseases

None of the 36 parameters proposed for evaluation in this section was considered highly important, 23 were considered moderately important, and all of them referred to uterine diseases immediately after calving (metritis and retained placenta), metabolic diseases, and dystocia. It is important to highlight that the most valued parameters within this category (rate 7) were those that consider these diseases in general, regardless of the lactation number of cows. Finally, nine parameters were considered of low importance and four irrelevant (Table 5). These results seem logical since the reproductive performance is being evaluated on a first visit to the farm context. Although the parameters included in this section could be used to monitor the constant evolution of the transition period and to partially forecast the immediate future reproductive performance of a dairy farm (13, 19, 29, 30), they do not provide a direct picture of the status and efficiency of the reproduction of a farm in a first visit.

**Table 5:** Answers given by consultants on Section 4: Postpartum and Metabolic Diseases, where 0 is of minimal or irrelevant importance and 10 represents the maximum importance.

**Postpartum and metabolic disease**
**parameter**	**Median**	**CI 95%**	**Interquartile range**	**Min–max**
Metritis, %	7.0	5.83–7.59	4.0	0–10
Retained placenta, %	7.0	5.56–7.41	4.5	0–10
Clinical ketosis, %	7.0	4.57–6.57	4.5	0–10
Clinical and subclinical ketosis, %	7.0	5.16–6.99	4.5	0–10
Metritis 1^st^ lact, %	7.0	4.32–6.36	6.5	0–10
Abomasal pathology, %	6.0	4.31–5.96	3.5	0–10
Hypocalcemia, %	6.0	5.34–7.06	4.0	0–10
Stillbirth, %	6.0	4.55–6.34	5.5	0–10
Hypocalcemia multiparous, %	6.0	4.10–6.17	6.5	0–10
Retained placenta multiparous, %	6.0	4.22–6.30	7.0	0–10
Metritis multiparous, %	6.0	4.12–6.16	7.5	0–10
Clinical and subclinical ketosis 1^st^ lact, %	6.0	3.89–5.90	8.0	0–10
Clinical and subclinical ketosis multiparous, %	6.0	4.13–6.27	8.0	0–10
Retained placenta 1^st^ lact, %	6.0	3.98–6.09	8.0	0–10
Twins, %	5.0	4.08–5.91	6.0	0–10
Dystocia, %	5.0	3.94–5.93	6.5	0–10
Clinical ketosis 1^st^ lact, %	5.0	3.15–5.13	7.0	0–10
Stillbirth multiparous, %	5.0	3.16–5.03	7.0	0–10
Hypocalcemia 1^st^ lact, %	5.0	3.16–5.20	7.0	0–10
Twins multiparous, %	5.0	3.28–5.07	7.0	0–10
Dystocia 1^st^ lact, %	5.0	3.57–5.56	7.0	0–10
Stillbirth 1^st^ lact, %	5.0	3.44–5.49	7.5	0–10
Clinical ketosis multiparous, %	5.0	3.37–5.56	8.0	0–10
Pyometra, %	4.0	2.92–4.86	6.5	0–10
Abomasal pathology 1^st^ lact, %	4.0	2.72–4.62	7.0	0–10
Abomasal pathology multiparous, %	4.0	2.82–4.68	7.0	0–9
Dystocia multiparous, %	4.0	3.04–4.91	7.0	0–10
Twins 1^st^ lact, %	3.0	2.24–4.04	5.5	0–10
Incorrect uterine involution after 30DIM, %	3.0	2.61–4.57	7.0	0–10
Perineal injury, %	2.0	2.06–3.85	5.5	0–9
Pyometra multiparous, %	2.0	2.12–3.92	6.0	0–8
Pyometra 1^st^ lact, %	2.0	2.17–4.02	6.0	0–10
Incorrect uterine involution after 30DIM 1^st^ lact, %	1.0	1.81–3.77	6.0	0–10
Incorrect uterine involution after 30DIM multiparous, %	1.0	1.84–3.78	6.0	0–10
Perineal injury multiparous, %	0.0	1.33–2.82	4.0	0–10
Perineal injury 1^st^ lact, %	0.0	1.66–3.35	5.0	0–10

*Results are expressed as median, CI 95%, interquartile range, and minimum (Min) and maximum (Max) values. Parameters are sorted according to the median obtained, from highest to lowest*.

*CI, Confidence interval 95%; DIM, Days in milk; lact, lactation*.

Consultants were grouped into five clusters in this section (see Supplementary Figure 5 in Supplementary Material). The proportion of each cluster was 30.6% (*n* = 15), 24.5% (*n* = 12), 22.4% (*n* = 11), 12.2% (*n* = 6), and 10.2% (*n* = 5) for clusters 1, 2, 3, 4, and 5, respectively. It is interesting to highlight that clusters 1 and 2 (20; 40.8% of the consultants) rated all parameters as highly or moderately important, except for 9 parameters classified as of low importance in cluster 1. In contrast, clusters 4 and 5 (23; 46.9% of the consultants) rated most parameters as of low importance or irrelevant (see Supplementary Table 4 in Supplementary Material for detailed data). This fact indicates a large difference of opinion between almost half of the respondents concerning the rest. Again, it could be explained because consultants are not used to asking for different data on the farm, depending on whether it is a first visit, which aims at a quick assessment of reproductive performance, or a routine visit, where they would need to monitor many more details through parameters that demonstrate causes of failure or improvements in the measures taken along the time.

Although none of the parameters in this section was categorized with mean values as highly important, when results were analyzed after hierarchical clustering, 53.1% of respondents (*n* = 26) (Clusters 1, 2, 3) considered the percentage of retained placenta in multiparous cows and the clinical and subclinical ketosis as highly important (see Supplementary Table 4 in Supplementary Material).

The distribution of consultants interviewed among the clusters in section 4 is affected by both, years of experience as a consultant in dairy reproduction (*P* = 0.01) and farm size (*P* = 0.003) (see Supplementary Figure 6 in Supplementary Material). Most consultants advising farms with 151 to 700 milking cows belong to clusters 1, 2, and 3 (72%). Clusters 1, 2, and 3 are the clusters were the most importance is given to uterine and metabolic diseases (see Supplementary Table 4 in Supplementary Material). In contrast, cluster 5 is composed of 50 and 41.6% of consultants with farms of >700 or <150 milking cows, respectively. This fact suggests that all consultants who do not consider postpartum diseases important to assess reproduction on a first visit advise smaller or larger farms. Regarding the years of experience of the consultants, consultants with 20 to 25 years of experience, are in clusters 1, 4, and 5. They either give great importance to uterine and metabolic diseases (6; 33.3% in Cluster 1), or they do not give any importance to them (33; 66.6% in clusters 4 and 5). It is also important to highlight that 69.2% of consultants (*n* = 34) with 15 to 20 years of experience consider uterine and postpartum diseases to be highly or moderately important and there is no consultant in this age group included in cluster 5, where all parameters of the section are considered irrelevant (see Supplementary Table 4 in Supplementary Material). There seems to be no middle ground for consultants with >15 years of experience regarding the value of postpartum and metabolic diseases. Consultants who consider themselves important are likely to use holistic approaches that englobe different sources of information during a first visit, maybe because they are convinced that there will be upcoming routine visits and already want to assess possible causes of reproductive problems in the future. It could also be that they did not differentiate between a first visit and a routine visit. Consultants who consider postpartum diseases information irrelevant on a first visit are aiming to assess the current reproductive performance situation, without delving deeper into possible past negative impacts, and extrapolating possible future problems in reproductive performance caused by postpartum diseases.

### Section 5: Heifers' Reproduction

This section included 50 parameters,seven were considered highly important (CR, first service CR, heat detection rate, % of anovulatory heifers, % heifers culled for reproductive reasons, age at first service, and age at first calving). Eighteen parameters were classified as moderately important, nine of low interest, and 16 resulted irrelevantly. Consultants rated age at first calving with 10 and a low interquartile range, being the most consistent and robust KPI for this section. Moreover, there was a high number of parameters considered irrelevant in this section. A reasonable explanation could be based on the redundancy of some of these parameters, among which only the age of the heifers differs. As an example, the survey includes a proportion of heifers >11, >12, >13, >14 months old not serviced (see Table 6).

**Table 6:** Answers given by consultants on Section 5: Heifers' Reproduction parameters, where 0 is of minimal or irrelevant importance and 10 represents the maximum importance.

**Heifers' reproduction parameters**	**Median**	**CI 95%**	**Interquartile range**	**Min–max**
Age at 1^st^ calving, d	10.0	7.79–9.30	2.5	0–10
CR, %	9.0	2.48–8.83	3.0	0–10
Heat detection rate, %	8.0	6.44–8.08	3.0	0–10
First service CR, %	8.0	6.53–8.36	3.0	0–10
Heifers culled for reproductive reason, %	8.0	5.57–7.48	4.0	0–10
Age at first service, d	8.0	6.31–8.17	4.0	0–10
Anovulatory heifers, %	8.0	3.57–5.77	8.0	0–10
Interval service to service, d	7.0	5.25–7.23	4.0	0–10
Services per pregnant heifer, *n*	7.0	5.78–7.72	4.5	0–10
Pregnancy loss, %	7.0	4.94–6.97	5.0	0–10
Culling rate heifers, %	7.0	5.28–7.15	5.0	0–10
Interval heat to heat, d	7.0	4.69–6.69	5.5	0–10
Pregnancy loss (1–90days), %	7.0	4.19–6.25	7.0	0–10
Heifers calving <23 months old, %	7.0	3.84–6.19	8.0	0–10
Abortion >90 days, %	7.0	4.17–6.36	9.0	0–10
Heifers calving <24 months old, %	7.0	4.53–6.97	10.0	0–10
CR of Inseminators, %	6.0	4.20–6.07	6.0	0–10
CR synchronized heifers, %	6.0	4.47–6.54	6.5	0–10
Heifers pregnant, %	6.0	3.58–5.76	8.0	0–10
Heifers >14 months old not serviced, %	6.0	3.82–6.05	8.0	0–10
Heifers/Cows, %	6.0	3.87–5.96	8.0	0–10
% “do not breed” heifers, %	6.0	3.96–6.03	8.0	0–10
Days at pregnancy diagnosis, d	5.0	4.07–5.96	5.0	0–10
Heifers <14 months old, %	5.0	2.83–4.83	7.0	0–10
21d. Pregnancy rate, %	5.0	5.10–7.30	7.5	0–10
Heifers >14 months old, %	4.0	2.64–4.66	7.0	0–10
CR of the sire, %	4.0	2.91–4.96	7.0	0–10
Heifers calving <22 months old, %	4.0	2.65–4.89	7.5	0–10
Open heifers > 16 months, %	4.0	3.21–5.73	9.0	0–10
Heifers >12 months old, %	3.0	2.43–4.34	6.0	0–10
Number of heifers, *n*	3.0	2.50–4.39	6.0	0–10
Heifers <12 months old, %	3.0	2.53–4.48	6.5	0–10
Ovarian cysts, %	2.0	2.12–4.15	6.0	0–10
Open heifers > 17 months, %	2.0	2.52–4.94	8.0	0–10
Heifers >11 months old not serviced, %	0.0	0.59–1.68	2.0	0–8
Open heifers > 12 months, %	0.0	0.79–2.38	2.0	0–10
Heifers >12 months old not serviced, %	0.0	0.94–2.31	4.0	0–8
Heifers >11 months old, %	0.0	0.96–2.46	4.0	0–10
Heifers <11 months old, %	0.0	1.13–2.74	4.0	0–10
Open heifers > 13 months, %	0.0	1.13–2.98	4.5	0–10
Heifers >13 months old, %	0.0	1.52–3.33	5.0	0–10
Heifers <13 months old, %	0.0	1.59–3.45	6.0	0–10
Heifers calving <21 months old, %	0.0	2.14–4.38	6.5	0–10
Heifers >13 months old not serviced, %	0.0	1.43–3.29	5.0	0–10
Heifers <2 standard deviations from 400 kg at 400 d, %	0.0	1.44–3.49	5.0	0–10
Heifer efficiency, %	0.0	1.78–3.88	7.0	0–10
Heifers <580 kg at calving, %	0.0	1.97–4.10	7.0	0–10
Early pregnancy loss (1–42 days), %	0.0	2.17–4.35	7.0	0–10
Open heifers > 14 months, %	0.0	2.24–4.57	7.5	0–10
Open heifers > 15 months, %	0.0	2.36–4.74	8.0	0–10

*Results are expressed as median, CI 95%, interquartile range and, minimum (Min) and maximum (Max) values. Parameters are sorted according to the median obtained, from highest to lowest*.

*CI, Confidence interval 95%; CR, Conception rate*.

Consultants surveyed preferred averages rather than proportions for the parameters with the highest scores. This can be explained for two reasons: consultants tend to use the most interesting parameters that can be directly obtained from the farm software (most farm software provides averages rather than proportions). The second reason could be that, classically, consultants have expressed these parameters as averages as they were easier to calculate, and producers are more useeeing see averages than medians. Anyway, the degree of spread around that average should also be considered, including the analysis of the standard deviation (31).

Answered surveys were grouped into five clusters for this section (Supplementary Figure 7 in Supplementary Material). Cluster 1 represented 32.2% of the consultants (n = 16) and 22 of the parameters were considered highly important and they are a combination of averages and proportions (i.e., age at first calving and % of cows calving before 23–24 months old). Cluster 2 included 28.6% of the consultants (n = 14) where 14 parameters were classified as highly important. Clusters 3 and 4 included the lowest percentage of consultants (5; 10.2 and 4; 8.2%, respectively) and considered 3 and 12 parameters as highly important, respectively. Lastly, cluster 5 represented 20% of the surveys answered (*n* = 10) and did not consider any of the parameters as highly important to evaluate the reproductive performance of a farm during a first visit (see Supplementary Table 5 in the Supplementary Material for detailed data). Clusters 1, 2, 3, and 4 agreed that CR and heat detection are highly important, while cluster 5 considered them moderately important. Following the same trend as in the case of cows, it is the consultant's priority to analyze the probability of getting a heifer pregnant. In the background, consultants from all clusters also consider important parameters related to the efficiency of the whole rearing process and the probability of achieving first the calving as soon as possible. In this case CR at first service, age at first calving, and 21d pregnancy rate are considered highly important (clusters 1, 2, and 4) or moderately important (clusters 3 and 5).

The CR and heat detection rate were the parameters evaluated as highly important by more participants (39; 79.6%), followed by first service CR, 21d pregnancy rate, age at first calving, and percentage of heifers calving <24 months old that were considered highly important by 69.4% of the consultants (*n* = 34) (see Table 7).

**Table 7:** Parameters evaluated as highly important (range 8–10) on Section 5: Heifers Reproduction, at least, 50% of the consultants surveyed after hierarchical clustering analysis.

**Heifers' reproduction**	**Median**	**CI 95%**	**Interquartile**	**Min–max**	**% of consultants that rated**	**Clusters mainly rating**
**parameter**			**range**		**the parameter as**	**the parameter as**
					**highly important**	**highly important**
Heat detection rate, %	8.0	6.44–8.08	3.0	0–10	79.6 (39)	1, 2, 3, 4
CR, %	9.0	2.48–8.83	3.0	0–10	79.6 (39)	1, 2, 3, 4
First service CR, %	8.0	6.53–8.36	3.0	0–10	69.4 (34)	1, 2, 4
Age at 1^st^ calving, d	10.0	7.79–9.30	2.5	0–10	69.4 (34)	1, 2, 4
Heifers culled for reproductive reason, %	8.0	5.57–7.48	4.0	0–10	61.2 (30)	1, 2

*Results are expressed as median, CI 95%, interquartile range, minimum (Min) and maximum (Max) values, and proportion of consultants that rated the parameter as highly important (number of observations within brackets). The clusters obtained after hierarchical clustering analysis where most of these highly important parameters were present are also depicted. Parameters are sorted according to the proportion of consultants that rated the parameter as highly important, from highest to lowest*.

*CI, Confidence interval 95%; CR, Conception rate*.

Following the line of assessment given in section 3 (Cows' reproduction), consultants considered it to be crucial to analyze data relating to the likelihood of getting heifers pregnant and having first calving at the right time. It is interesting to note, however, that the parameter rated as the most important is the age at first calving. We consider this to be logical and important, as this parameter not only evaluates the reproductive efficiency of the more recent past, but also the entire young stock rearing process. However, a much better KPIs for heifers' reproductive assessment (with a lesser lag) would be the age at conception but unfortunately, this item was not shown in the survey due to a technical problem. The heifer rearing process deserves to be considered because it is a long-term investment for the farms and has a sufficiently large impact on the productive future of the farm (32–34).

The distribution of consultants among the different clusters in this section has not been affected either by the size of the farm (*P* = 0.37) or by the consultants' years of experience (*P* = 0.19). Unlike the other sections, it is evident that in the case of heifers, all consultants, regardless of the size of the farm they are advising and years of experience, agree that the relevant parameters are those with a direct effect on the optimal age at 1^st^ calving.

## Conclusions

Consultants never use only one primary KPI to evaluate any of the presented sections, as they use several of them instead (in variable quantitative range). Despite the presence of a great number of parameters likely to be KpI to assess the reproductive performance of a farm, consultants clearly prefer parameters that provide information about heat detection, fertility, and when the pregnancy is achieved regarding the production cycle of a dairy cow. Consultants also show high interest in getting a general overview of milk production, the farming efficiency, and the heifer-rearing process, but the farm size and the years of experience of the consultant is other factor influencing in the type and numbers of parameters chosen to be KPI.

The parameters that consultants specialized in dairy reproduction, which are mostly considered as primary KPI, to assess the reproductive status of a conventional dairy farm in a first visit are:

General data of the farm: Culling rate (%), pregnant cows (%), average DIM (d), cows culled for reproductive reason (%), 305-d yield (Kg), herd status for BVDV (present/absent), and % of 1^st^ lactation cows in the herd.

Cows' reproduction: First service CR (%), overall pregnancy rate (%), voluntary waiting period (d), CR (%), heat detection rate (%), days open (d), cows not pregnant >200 DIM (%), 21d. pregnancy rate (%), CR of the first service in 1st lactation cows, CR of the first service in multiparous cows (%), CR of the 1st lactation cows (%), CR of the multiparous cows (%), calving to first service interval (d), CR synchronized cows (%), services per pregnancy, percentage abortion in >90 days, and percent conceiving of served (%).

Heifers' reproduction: Heat detection rate (%), CR (%), CR of the first service (%), age at first calving (d), and heifers culled for reproductive reason (%).

The parameters rated with the highest importance (rate 10) that could be considered for an easy, fast, and universal use in a first visit to assess the reproductive status were: First service CR (%), overall pregnancy rate (%) and 21d pregnancy rate (%) for cows and age at first calving (d) for heifers. Lastly, parameters that monitor postpartum and metabolic diseases were not considered as highly important (range 8–10) to evaluate the reproductive performance of a farm in a first visit.

Authors have foreseen to carry out a follow-up study to decipher if the best KPI set up in this research work are also useful for routine visits on a dairy farm using the same approach.

## Data Availability Statement

The raw data supporting the conclusions of this article will be made available by the authors, without undue reservation.

## Ethics Statement

The animal study was reviewed and approved by Ethic Committe of the University of Lleida.

## Author Contributions

RA: conceptualization, methodology, investigation, writing—original draft, and visualization. LF: formal analysis, methodology, validation, writing—original draft, and visualization. AB: methodology, validation, visualization, and writing—review and editing. All authors contributed to the article and approved the submitted version.

## Conflict of Interest

The authors declare that the research was conducted in the absence of any commercial or financial relationships that could be construed as a potential conflict of interest.

## Publisher's Note

All claims expressed in this article are solely those of the authors and do not necessarily represent those of their affiliated organizations, or those of the publisher, the editors and the reviewers. Any product that may be evaluated in this article, or claim that may be made by its manufacturer, is not guaranteed or endorsed by the publisher.
