# Supplementary material for: Key Performance Indicators Used by Dairy Consultants During the Evaluation of Reproductive Performance in a First Visit
Source: Front Vet Sci. 2022 Jun 23;9:871079. doi: 10.3389/fvets.2022.871079 (PMC9260265; doi:10.3389/fvets.2022.871079)
Supplement: Supplementary file 1 [file Table_1.DOCX]

Supplementary Table 1. Content of the survey sent in Google Forms format to the participants. The table includes the section, the parameter, the exact definition and the type of answer that consultants had to fulfill.

| SECTION | PARAMETER | DEFINITION | ANSWEAR |
| --- | --- | --- | --- |
| Section 1 Consultant and farm model |  |  |  |
|  | Country | Country were the consultant mostly works | Text |
|  | Age | Age of the consultant | 20 to 30  31 to 40  41 to 50  51 to 60  61 to 70 |
|  | Years of Experience | Years of experience consulting for reproduction in dairy farms | 0 to 5  5 to 10  10 to 15  15 to 20  20 to 25  >25 |
|  | Farm System | Most common farm system that consultant advises | Pasture at any time  Never Pastures |
|  | Cows Breed | Most common breed present in the farms that consultant advises | Text |
|  | Main Breeding system | Most common breeding technique used in the farms that consultant advises | Mating  Artificial Insemination  Embryo transfer  In vitro fertilization |
|  | Calving Season | Farms with the goal of having one or two calving seasons or not (homogeneous number of calvings during the year) | Yes  No |
|  | Type of stall | Type of allocation that cows have in the stall | Tied  Cubicles/Free Stall  Bedded Pack |
|  | Number of milkings/ day | Number of times that cows are milked | 2  3 |
|  | Size of the Farm | Numbers of milking cows that farms advised have. | 1 to 50  51-150  151-300  301-500  501-700  701-1000  1001-3000  >3000 |
|  | Close up pen |  | Yes  No |
|  | Time span of data requested | Period of time that data requested by the consultant in the first visit to the farm would cover. | Current Year  Last 3 months  Last 6 months  Last year  Last 3 years  Last 5 years  Last 10 years |
| Section 2 General data of the farm |  |  |  |
|  | Average number of lactation | Average of all lactation numbers of cows during the period | 0 1 2 3 4 5 6 7 8 9 10 |
|  | Total number of cows | Average number of cows present in the farm during the period | 0 1 2 3 4 5 6 7 8 9 10 |
|  | Number of milking cows | Average number of milking cows during the period | 0 1 2 3 4 5 6 7 8 9 10 |
|  | Number of 1^st^ lactation cows | Average number of 1^st^ lactation milking cows during the period | 0 1 2 3 4 5 6 7 8 9 10 |
|  | % 1^st^ lactation cows | Average number of 1^st^ lactation milking cows divided by the total number of milking cows during the period | 0 1 2 3 4 5 6 7 8 9 10 |
|  | % Dry cows | Average number of dry off cows divided by the total number of cows present in the farm during the period | 0 1 2 3 4 5 6 7 8 9 10 |
|  | Number of dry cows | Average number of cows that are dry during the period | 0 1 2 3 4 5 6 7 8 9 10 |
|  | Monthly milk yield | Average production of kilos of milk produced every month | 0 1 2 3 4 5 6 7 8 9 10 |
|  | Daily milk yield | Average production of kilos of milk produced daily | 0 1 2 3 4 5 6 7 8 9 10 |
|  | Lactating cows daily milk yield | Average of the total kilos of milk produced in one day divided by for the number of milking cows | 0 1 2 3 4 5 6 7 8 9 10 |
|  | All cows daily milk yield | Average of the total kilos of milk produced in one day divided by the total (both milking and dry) number of cows present in the farm | 0 1 2 3 4 5 6 7 8 9 10 |
|  | Total number of pregnant cows | Average total number of pregnant cows during the period | 0 1 2 3 4 5 6 7 8 9 10 |
|  | % of pregnant cows | Average total number of pregnant cows divided by the total number of cows present in the farm during the period | 0 1 2 3 4 5 6 7 8 9 10 |
|  | Days in milk (DIM) | Average days in milk for all cows during the period | 0 1 2 3 4 5 6 7 8 9 10 |
|  | Average days dry | Average days in dry off for all cows during the period | 0 1 2 3 4 5 6 7 8 9 10 |
|  | 305 day yield | Average cumulative production by day 305 of lactation for all cows during the period. | 0 1 2 3 4 5 6 7 8 9 10 |
|  | Culling rate | Average number of cows culled, sold, dead or transferred out of the herd divided by the average number of total cows (both milking and dry) in the same period. | 0 1 2 3 4 5 6 7 8 9 10 |
|  | Failure to conceive culling rate | Average number of cows culled due to a failure to conceive as a percentage of the total number of eligible cows calving in the period. | 0 1 2 3 4 5 6 7 8 9 10 |
|  | % "do not breed" cows | Average number of cows with decision of "do not breed" divided by the total number of cows in the same period | 0 1 2 3 4 5 6 7 8 9 10 |
|  | % Cows culled for reproductive reason | Average number culled cows for reproductive reasons divided by the total number cows culled during the period | 0 1 2 3 4 5 6 7 8 9 10 |
|  | % Cows culled for lameness reason | Average number culled cows for lameness reasons divided by the total number cows culled during the period | 0 1 2 3 4 5 6 7 8 9 10 |
|  | % Cows culled for mastitis reason | Average number culled cows for mastitis reasons divided by the total number cows culled during the period | 0 1 2 3 4 5 6 7 8 9 10 |
|  | % Cows culled for accident reason | Average number culled cows due to accidents divided by the total number cows culled during the period | 0 1 2 3 4 5 6 7 8 9 10 |
|  | Average somatic cell count (SCC) | Average SCC of all milk samples taken in the last 12 months. | 0 1 2 3 4 5 6 7 8 9 10 |
|  | % Clinical mastitis | Average number of clinical mastitis cases divided by the total number cows lactation during the period | 0 1 2 3 4 5 6 7 8 9 10 |
|  | % Lameness | Average number of lameness cases divided by the total number of cows (both milking and dry) during the period | 0 1 2 3 4 5 6 7 8 9 10 |
|  | Peak milk 1^st^ lactation cows | Average of highest level of milk in kg that 1^st^ lactation cows produce during the 1^st^ lactation. | 0 1 2 3 4 5 6 7 8 9 10 |
|  | Peak milk 2^nd^ lactation cows | Average of highest level of milk in kg that 2^nd^ lactation cows produce during the 2^nd^ lactation. | 0 1 2 3 4 5 6 7 8 9 10 |
|  | Peak milk 3^rd^ lactation cows | Average of highest level of milk in kg that 3^rd^ lactation cows produce during the 3^rd^ lactation. | 0 1 2 3 4 5 6 7 8 9 10 |
|  | Peak milk >3^rd^ lactation cows | Average of highest level of milk in kg that cows with more than three lactations produce during their present lactation. | 0 1 2 3 4 5 6 7 8 9 10 |
|  | Peak milk (DIM) 1^st^ lactation cows | Average of days in milk that 1^st^ lactation cows had to reach peak milk. | 0 1 2 3 4 5 6 7 8 9 10 |
|  | Peak milk (DIM) 2^nd^ lactation cows | Average of days in milk that 2^nd^ lactation cows had to reach the peak milk. | 0 1 2 3 4 5 6 7 8 9 10 |
|  | Peak milk (DIM) 3^rd^ lactation cows | Average of days in milk that 3^rd^ lactation cows had to reach the peak milk. | 0 1 2 3 4 5 6 7 8 9 10 |
|  | Peak milk (DIM) >3^rd^ lactation cows | Average of days in milk that cows with more than three lactations had to reach the peak milk. | 0 1 2 3 4 5 6 7 8 9 10 |
|  | % Replacement | Average number of heifers or purchased cows that go into "milking cow" status for the first time in that herd divided for the total number of cows (both lactating and dry) during the period | 0 1 2 3 4 5 6 7 8 9 10 |
|  | Average lactation number of culled cows | Average lactation number of cows culled in the farm during the period | 0 1 2 3 4 5 6 7 8 9 10 |
|  | Total number of cows culled | Average number of cows culled in the farm during the period | 0 1 2 3 4 5 6 7 8 9 10 |
|  | Average DIM culled cows | Average days in milk for all cows culled during the period | 0 1 2 3 4 5 6 7 8 9 10 |
|  | Herd status for Brucellosis | Herd status (Positivity and/or Prevalence) for *Brucella spp* | Present  Absent |
|  | Herd status for Neosporosis | Herd status (Positivity and/or Prevalence) for *Neospora caninum* | Present  Absent |
|  | Herd status for BVDV | Herd status (Positivity and/or Prevalence) for Bovine Viral Diarrhoea Virus | Present  Absent |
|  | Herd status for IBR-IPV | Herd status (Positivity and or Prevalence) for Infectious Bovine Infectious Rhinotracheitis-Infectious Pustular Vulvovaginitis-Infectious Balanitis caused by Bovine Herpesvirus | Present  Absent |
|  | Herd status for FMD | Herd status (Positivity and/or Prevalence) for Foot and Mouth Disease caused by Aftovirus | Present  Absent |
|  | Other parameters useful for you | Other parameters that the consultant uses and not included in the survey | Text |
| Section 3 Cow reproduction |  |  |  |
|  | Voluntary waiting period | Average number of postpartum days that cows are deliberately left unserved | 0 1 2 3 4 5 6 7 8 9 10 |
|  | % conceiving of served | Average proportion of the total number of animals served that conceive | 0 1 2 3 4 5 6 7 8 9 10 |
|  | Overall pregnancy rate | Average number of pregnancies divided by the total number of eligible services | 0 1 2 3 4 5 6 7 8 9 10 |
|  | First service conception rate | Average number of pregnancies divided by the total number of first services. | 0 1 2 3 4 5 6 7 8 9 10 |
|  | Days open | Average number of days from calving to conception for those cows conceiving and from calving to culling for those failing to conceive | 0 1 2 3 4 5 6 7 8 9 10 |
|  | Days to culling | Average number of day between conception and culling | 0 1 2 3 4 5 6 7 8 9 10 |
|  | Non- return rate | Average number of cows not returning to service as a percentage of the total number of cows served | 0 1 2 3 4 5 6 7 8 9 10 |
|  | Conception rate | Average number of pregnancies divided by the total number of services | 0 1 2 3 4 5 6 7 8 9 10 |
|  | % Ovarian cysts | Average number of cows diagnosed with an ovarian cyst divided by the total number of eligible cows in the herd during the period | 0 1 2 3 4 5 6 7 8 9 10 |
|  | % Anovulatory cows | Average number of cows diagnosed as anovulatory divided by the total number of eligible cows during the period | 0 1 2 3 4 5 6 7 8 9 10 |
|  | 21d Pregnancy rate | Average number of pregnancies divided by the number of eligible cycles over a 21 days during the evaluation period | 0 1 2 3 4 5 6 7 8 9 10 |
|  | Services per pregnancy | Average total number of services over the total number of pregnant cows during the period | 0 1 2 3 4 5 6 7 8 9 10 |
|  | % Pregnancy loss | Average number of pregnant cows that lost pregnancy divided by the total number of pregnant cows during the period | 0 1 2 3 4 5 6 7 8 9 10 |
|  | % Early pregnancy loss (1-42 days) | Average number of pregnant cows that lost pregnancy during first 42 days of gestation divided by the total number of pregnant during the period | 0 1 2 3 4 5 6 7 8 9 10 |
|  | % Pregnancy loss( 1-90days) | Average number of pregnant cows that lost pregnancy during the first 90 d of gestation divided by the total number of pregnancies during the period | 0 1 2 3 4 5 6 7 8 9 10 |
|  | % Abortion >90 days | Average number of pregnant cows that lost pregnancy beyond 90 d of gestation divided by the total number of pregnant cows during the period | 0 1 2 3 4 5 6 7 8 9 10 |
|  | % Conception rate synchronized cows | Average number of pregnant cows from one hormonal treatment divided by the total number of services conducted after a hormonal treatment | 0 1 2 3 4 5 6 7 8 9 10 |
|  | Conception rate of the sire | Average number of pregnant cows from one sire divided by the total number of services of the same sire during the period | 0 1 2 3 4 5 6 7 8 9 10 |
|  | Conception rate of inseminators | Average number of pregnant cows serviced by one technician divided by the total number of services that the same technician performed during the period | 0 1 2 3 4 5 6 7 8 9 10 |
|  | Days at pregnancy diagnosis | Average interval (in days) between service and pregnancy diagnosis | 0 1 2 3 4 5 6 7 8 9 10 |
|  | % Conception rate first service in 1^st^ lactation cows | Average number of 1^st^ lactation cows pregnant at first service divided by the total number of 1^st^ lactation cows that were first serviced during the period | 0 1 2 3 4 5 6 7 8 9 10 |
|  | % Conception rate first service in 2^nd^ lactation cows | Average number of 2^nd^ lactation cows pregnant at first service divided by the total number of 2^nd^ lactation cows that were first serviced during the period | 0 1 2 3 4 5 6 7 8 9 10 |
|  | % Conception rate first service in 3^rd^ lactation cows | Average number of 3^rd^ lactation cows pregnant at first service divided by the total number of 3^rd^ lactation cows that were first serviced during the period | 0 1 2 3 4 5 6 7 8 9 10 |
|  | % Conception rate first service in >3^rd^ lactation cows | Average number of cows with more than three lactations pregnant at first service divided by the total number cows with more than lactations first serviced during the period | 0 1 2 3 4 5 6 7 8 9 10 |
|  | % Conception rate first service in multiparous cows | Average number of multiparous cows pregnant at first service divided by the total number of multiparous cows first serviced during the period | 0 1 2 3 4 5 6 7 8 9 10 |
|  | % Conception rate 1^st^ lactation cows | Average number of 1^st^ lactation cows pregnant divided by the total number 1^st^ lactation cows of serviced during the period | 0 1 2 3 4 5 6 7 8 9 10 |
|  | % Conception rate 2^nd^ lactation cows | Average number of 2^nd^ lactation cows pregnant divided by the total number of 2^nd^ lactation cows serviced during the period | 0 1 2 3 4 5 6 7 8 9 10 |
|  | % Conception rate 3^rd^ lactation cows | Average number of 3^rd^ lactation cows pregnant divided by the total number of 3^rd^ lactation cows serviced during the period | 0 1 2 3 4 5 6 7 8 9 10 |
|  | % Conception rate >3^rd^ lactation cows | Average number of cows with more than three lactations pregnant divided by the total number of cows with more than three lactations serviced during the period | 0 1 2 3 4 5 6 7 8 9 10 |
|  | % Conception rate multiparous cows | Average number of multiparous cows pregnant divided by the total number multiparous cows serviced during the period | 0 1 2 3 4 5 6 7 8 9 10 |
|  | Submission rate first 3 weeks | Average percentage of cows receiving at least one insemination during the first three weeks of the breeding period – which begins once the Voluntary Waiting Period has been completed. | 0 1 2 3 4 5 6 7 8 9 10 |
|  | Submission rate | Average number of cows in the herd receiving a first service | 0 1 2 3 4 5 6 7 8 9 10 |
|  | Calving to first service interval | Average interval (in days) between calving and first service | 0 1 2 3 4 5 6 7 8 9 10 |
|  | Interval heat to heat | Average number of days between consecutive heats | 0 1 2 3 4 5 6 7 8 9 10 |
|  | Interval service to service | Average number of days between consecutive services | 0 1 2 3 4 5 6 7 8 9 10 |
|  | Heat detection rate | Average number of heats identified divided by the number of eligible cycles during an eligible period | 0 1 2 3 4 5 6 7 8 9 10 |
|  | 2–17 day return to service/heat, % | Average proportion of eligible cows that return to estrous within 2 to 17 days | 0 1 2 3 4 5 6 7 8 9 10 |
|  | 18–24 day return to service/heat, % | Average proportion of eligible cows that return to estrous within 18 to 24 days | 0 1 2 3 4 5 6 7 8 9 10 |
|  | 25–35 day return to service/heat, % | Average proportion of eligible cows that return to estrous within 25 to 35 days | 0 1 2 3 4 5 6 7 8 9 10 |
|  | 36–48 day return to service/heat, % | Average proportion of eligible cows that return to estrous within 36 to 48 days | 0 1 2 3 4 5 6 7 8 9 10 |
|  | >49 day return to service/heat, % | Average proportion of eligible cows that return to estrous after 49 days | 0 1 2 3 4 5 6 7 8 9 10 |
|  | 100-Day In-calf rate | Average percentage of cows in the herd confirmed pregnant within 100 days of calving | 0 1 2 3 4 5 6 7 8 9 10 |
|  | % Cows served <90 DIM | Average proportion of cows that are served before 90 days in milk | 0 1 2 3 4 5 6 7 8 9 10 |
|  | Herd calving to conception interval | Average number of days from calving to the service at which a cow becomes pregnant | 0 1 2 3 4 5 6 7 8 9 10 |
|  | Calving interval | Average number of days elapsed between current and previous calving | 0 1 2 3 4 5 6 7 8 9 10 |
|  | % Cows not pregnant >200 DIM | Average proportion of cows that are not pregnant after day 200 in milk | 0 1 2 3 4 5 6 7 8 9 10 |
|  | % Cows not pregnant >150 DIM | Average proportion of cows that are not pregnant after day 150 in milk | 0 1 2 3 4 5 6 7 8 9 10 |
|  | Calvings per month | Average of the number of calvings every month | 0 1 2 3 4 5 6 7 8 9 10 |
|  | % 1^st^ lactation cows calved | Average number of heifers that calved divided by the total number of cows that calved | 0 1 2 3 4 5 6 7 8 9 10 |
|  | OTHER PARAMETERS USEFUL FOR YOU | Other parameters that the consultant uses and not included in the survey | Text |
| Section 4 Postpartum and metabolic diseases |  |  |  |
|  | % Metritis | Average number of cows with metritis divided by the total number of cows that calved during the period | 0 1 2 3 4 5 6 7 8 9 10 |
|  | % Retained placenta | Average number of cows with retained placenta divided by the total number of cows that calved during the period | 0 1 2 3 4 5 6 7 8 9 10 |
|  | % Clinical ketosis | Average number of cows with clinical ketosis divided by the total number of cows that calved during the period | 0 1 2 3 4 5 6 7 8 9 10 |
|  | % Clinical and subclinical ketosis | Average number of cows with subclinical and clinical ketosis divided by the total number of cows that calved during the period | 0 1 2 3 4 5 6 7 8 9 10 |
|  | % Hypocalcaemia | Average number of cows with hypocalcaemia divided by the total number of cows that calved during the period | 0 1 2 3 4 5 6 7 8 9 10 |
|  | % Stillbirth | Average number of cows that delivered a dead calf or the calf died within the first 24 hours divided by the total number of cows that calved during the period | 0 1 2 3 4 5 6 7 8 9 10 |
|  | % Twins | Average number of cows with twins parturition divided by the total number of cows that calved during the period | 0 1 2 3 4 5 6 7 8 9 10 |
|  | % Dystocia | Average number of cows with dystocia divided by the total number of cows that calved | 0 1 2 3 4 5 6 7 8 9 10 |
|  | % Metritis 1^st^ lactation cows | Average number of 1^st^ lactation cows with metritis divided by the total number of 1^st^ lactation cows that calved | 0 1 2 3 4 5 6 7 8 9 10 |
|  | % Retained placenta 1^st^ lactation cows | Average number of 1^st^ lactation cows with retained placenta divided by the total number of 1^st^ lactation cows that calved | 0 1 2 3 4 5 6 7 8 9 10 |
|  | % Clinical ketosis 1^st^ lactation cows | Average number of 1^st^ lactation cows with clinical ketosis divided by the total number of 1^st^ lactation cows that calved | 0 1 2 3 4 5 6 7 8 9 10 |
|  | % Clinical and subclinical ketosis 1^st^ lactation cows | Average number of 1^st^ lactation cows with subclinical and clinical ketosis divided by the total number of 1^st^ lactation cows that calved | 0 1 2 3 4 5 6 7 8 9 10 |
|  | % Hypocalcaemia 1^st^ lactation cows | Average number of 1^st^ lactation cows with hypocalcaemia divided by the total number of 1^st^ lactation cows that calved | 0 1 2 3 4 5 6 7 8 9 10 |
|  | % Stillbirth 1^st^ lactation cows | Average number of 1^st^ lactation cows that delivered a dead calf or the calf died within the first 24 hours divided by the total number of 1^st^ lactation cows that calved | 0 1 2 3 4 5 6 7 8 9 10 |
|  | % Twins 1^st^ lactation cows | Average number of 1^st^ lactation cows with twins parturition divided by the total number of 1^st^ lactation cows that calved | 0 1 2 3 4 5 6 7 8 9 10 |
|  | % Dystocia 1^st^ lactation cows | Average number of 1^st^ lactation cows with dystocia divided by the total number of 1^st^ lactation cows that calved | 0 1 2 3 4 5 6 7 8 9 10 |
|  | % Metritis multiparous | Average number of multiparous cows with metritis divided by the total number of multiparous cows that calved | 0 1 2 3 4 5 6 7 8 9 10 |
|  | % Retained placenta multiparous | Average number of multiparous cows with retained placenta divided by the total number of multiparous cows that calved | 0 1 2 3 4 5 6 7 8 9 10 |
|  | % Clinical ketosis multiparous | Average number of multiparous cows with clinical ketosis divided by the total number of multiparous cows that calved | 0 1 2 3 4 5 6 7 8 9 10 |
|  | % Clinical and subclinical ketosis multiparous | Average number of multiparous cows with subclinical and clinical ketosis divided by the total number of multiparous cows that calved | 0 1 2 3 4 5 6 7 8 9 10 |
|  | % Hypocalcaemia multiparous | Average number of multiparous cows with hypocalcaemia divided by the total number of multiparous cows that calved | 0 1 2 3 4 5 6 7 8 9 10 |
|  | % Stillbirth multiparous | Average number of multiparous cows that delivered a dead calf or the calf died within the first 24 hours divided by the total number of multiparous cows that calved | 0 1 2 3 4 5 6 7 8 9 10 |
|  | % Twins multiparous | Average number of multiparous cows with twins parturition divided by the total number of multiparous cows that calved | 0 1 2 3 4 5 6 7 8 9 10 |
|  | % Dystocia multiparous | Average number of multiparous cows with dystocia divided by the total number of multiparous cows that calved | 0 1 2 3 4 5 6 7 8 9 10 |
|  | % Incorrect uterine involution after 30DIM | Average number of cows with an incorrect uterine involution after day thirty in milk divided by the total number of cows that calved | 0 1 2 3 4 5 6 7 8 9 10 |
|  | % Incorrect uterine involution after 30DIM 1^st^ lactation cows | Average number of 1^st^ lactation cows with an incorrect uterine involution after day thirty in milk divided by the total number of 1^st^ lactation cows that calved | 0 1 2 3 4 5 6 7 8 9 10 |
|  | % Incorrect uterine involution after 30DIM multiparous | Average number of multiparous cows with an incorrect uterine involution after day thirty in milk divided by the total number of multiparous cows that calved | 0 1 2 3 4 5 6 7 8 9 10 |
|  | % Pyometra | Average number of cows with pyometra divided by the total number of cows that calved | 0 1 2 3 4 5 6 7 8 9 10 |
|  | % Pyometra 1^st^ lactation | Average number of 1^st^ lactation cows with pyometra divided by the total number of 1^st^ lactation cows that calved | 0 1 2 3 4 5 6 7 8 9 10 |
|  | % Pyometra multiparous | Average number of multiparous cows with pyometra divided by the total number of multiparous cows that calved | 0 1 2 3 4 5 6 7 8 9 10 |
|  | % Perineal injury | Average number of cows with perineal injury divided by the total number of cows that calved | 0 1 2 3 4 5 6 7 8 9 10 |
|  | % Perineal injury 1^st^ lactation | Average number of 1^st^ lactation cows with perineal injury divided by the total number of 1^st^ lactation cows that calved | 0 1 2 3 4 5 6 7 8 9 10 |
|  | % Perineal injury multiparous | Average number of multiparous cows with perineal injury divided by the total number of multiparous cows that calved | 0 1 2 3 4 5 6 7 8 9 10 |
|  | % Abomasal pathology | Average number of cows diagnosed with abomasal pathology divided by the total number of cows calved | 0 1 2 3 4 5 6 7 8 9 10 |
|  | % Abomasal pathology 1^st^ lactation | Average number of 1^st^ lactation cows diagnosed with abomasal pathology divided by the total number of 1^st^ lactation cows calved | 0 1 2 3 4 5 6 7 8 9 10 |
|  | % Abomasal pathology multiparous | Average number of multiparous cows diagnosed with abomasal pathology divided by the total number of multiparous cows calved | 0 1 2 3 4 5 6 7 8 9 10 |
|  | OTHER PARAMETERS USEFUL FOR YOU | Other parameters that the consultant uses and not included in the survey | Text |
| Section 5 Heifer reproduction |  |  |  |
|  | Conception rate | Average number of pregnancies in heifers divided by the total number of services in heifers during the period | 0 1 2 3 4 5 6 7 8 9 10 |
|  | First service conception rate | Average number of heifers pregnant at first service divided by the total number of heifers that were first serviced during the period | 0 1 2 3 4 5 6 7 8 9 10 |
|  | Interval heat to heat | Average number of days between consecutive heats | 0 1 2 3 4 5 6 7 8 9 10 |
|  | Interval service to service | Average number of days between consecutive services | 0 1 2 3 4 5 6 7 8 9 10 |
|  | Heat detection rate | Average number of heats identified divided by the number of eligible cycles during the period | 0 1 2 3 4 5 6 7 8 9 10 |
|  | % Ovarian Cysts | Average number of eligible heifers diagnosed with an ovarian cyst divided by the total number of eligible heifers to be serviced in the herd. | 0 1 2 3 4 5 6 7 8 9 10 |
|  | % Anovulatory heifers | Average number of eligible heifers diagnosed as anovulatory divided by the total number of eligible heifers to be serviced in the herd | 0 1 2 3 4 5 6 7 8 9 10 |
|  | 21d Pregnancy rate | Average number of heifers that became pregnant divided by the number of heifers eligible over a 21-day period | 0 1 2 3 4 5 6 7 8 9 10 |
|  | Services per pregnant heifer | Average number of services in heifers over the total number of pregnant heifers during the period | 0 1 2 3 4 5 6 7 8 9 10 |
|  | Culling rate heifers | Average number of heifers that are sold, die or are transferred out of the herd before having the first parturition in the period divided by the total number of heifers | 0 1 2 3 4 5 6 7 8 9 10 |
|  | % Heifers culled for reproductive reason | Average number culled heifers for reproductive reasons divided by the total number heifers culled | 0 1 2 3 4 5 6 7 8 9 10 |
|  | % "do not breed" heifers | Average number of heifers with decision of "do not breed" divided by the total number of heifers | 0 1 2 3 4 5 6 7 8 9 10 |
|  | Age at first service | Average age at which heifers were serviced for the first time | 0 1 2 3 4 5 6 7 8 9 10 |
|  | Age at first calving | Average age at which heifers calved for the first time | 0 1 2 3 4 5 6 7 8 9 10 |
|  | % Heifers calving <24 months old | Average proportion of heifers that calved younger than 24 months | 0 1 2 3 4 5 6 7 8 9 10 |
|  | % Heifers calving <23 months old | Average proportion of heifers that calved younger than 23 months | 0 1 2 3 4 5 6 7 8 9 10 |
|  | % Heifers calving <22 months old | Average proportion of heifers that calved younger than 22 months | 0 1 2 3 4 5 6 7 8 9 10 |
|  | % Heifers calving <21 months old | Average proportion of heifers that calved younger than 21 months | 0 1 2 3 4 5 6 7 8 9 10 |
|  | Conception rate synchronized heifers | Average number of pregnant heifers from one hormonal treatment divided by the number of services in heifers using a hormonal treatment. | 0 1 2 3 4 5 6 7 8 9 10 |
|  | Conception rate of the sire | Average number of pregnant heifers from one sire divided by the total number of services of the same sire. | 0 1 2 3 4 5 6 7 8 9 10 |
|  | Conception rate of inseminators | Average number of pregnant heifers from one technician divided by the total number of services performed by that technician | 0 1 2 3 4 5 6 7 8 9 10 |
|  | Days at pregnancy diagnosis | Average interval (in days) between service and pregnancy diagnosis | 0 1 2 3 4 5 6 7 8 9 10 |
|  | Number of heifers | Average number of heifers present in the herd | 0 1 2 3 4 5 6 7 8 9 10 |
|  | % heifers/cows | Average number of heifers divided by the number of cows present in the herd | 0 1 2 3 4 5 6 7 8 9 10 |
|  | % of heifers <14 months old | Average proportion (over total number of heifers) of heifers younger than 14 months | 0 1 2 3 4 5 6 7 8 9 10 |
|  | % of heifers >14 months old | Average proportion (over total number of heifers) older than 14 months | 0 1 2 3 4 5 6 7 8 9 10 |
|  | % of heifers <13 months old | Average proportion (over total number of heifers) of heifers younger than 13 months | 0 1 2 3 4 5 6 7 8 9 10 |
|  | % of heifers >13 months old | Average proportion (over total number of heifers) of heifers older than 13 months | 0 1 2 3 4 5 6 7 8 9 10 |
|  | % of heifers <12 months old | Average proportion (over total number of heifers) of heifers younger than 12 months | 0 1 2 3 4 5 6 7 8 9 10 |
|  | % of heifers >12 months old | Average proportion (over total number of heifers) of heifers older than 12 months | 0 1 2 3 4 5 6 7 8 9 10 |
|  | % of heifers <11 months old | Average proportion (over total number of heifers) of heifers younger than 11 months | 0 1 2 3 4 5 6 7 8 9 10 |
|  | % of heifers >11 months old | Average proportion (over total number of heifers) of heifers older than 11 months | 0 1 2 3 4 5 6 7 8 9 10 |
|  | % Heifers >14 months old not serviced | Average proportion of heifers older than 14 months that are not served | 0 1 2 3 4 5 6 7 8 9 10 |
|  | % Heifers >13 months old not serviced | Average proportion of heifers older than 13 months that are not served | 0 1 2 3 4 5 6 7 8 9 10 |
|  | % Heifers >12 months old not serviced | Average proportion of heifers older than 12 months that are not served | 0 1 2 3 4 5 6 7 8 9 10 |
|  | % Heifers >11 months old not serviced | Average proportion of heifers older than 11 months that are not served | 0 1 2 3 4 5 6 7 8 9 10 |
|  | % Heifers pregnant | Average proportion of heifers that are pregnant | 0 1 2 3 4 5 6 7 8 9 10 |
|  | % Pregnancy loss | Average number of pregnant heifers that lost pregnancy divided by the total number of pregnant heifers | 0 1 2 3 4 5 6 7 8 9 10 |
|  | % Early pregnancy loss (1-42 days) | Average number pregnant heifers that lost 1-42 days pregnancy divided by the total number of pregnant heifers | 0 1 2 3 4 5 6 7 8 9 10 |
|  | % Pregnancy loss( 1-90days) | Average number pregnant heifers that lost 1-90 days pregnancy divided by the total number of pregnant heifers | 0 1 2 3 4 5 6 7 8 9 10 |
|  | % Abortion >90 days | Average number pregnant heifers that lost more than 90 days pregnancies divided by the total number of pregnant heifers | 0 1 2 3 4 5 6 7 8 9 10 |
|  | % of open heifers > 12 months | Average proportion of heifers older than 12 months that are not pregnant | 0 1 2 3 4 5 6 7 8 9 10 |
|  | % of open heifers > 13 months | Average proportion of heifers older than 13 months that are not pregnant | 0 1 2 3 4 5 6 7 8 9 10 |
|  | % of open heifers > 14 months | Average proportion of heifers older than 14 months that are not pregnant | 0 1 2 3 4 5 6 7 8 9 10 |
|  | % of open heifers > 15 months | Average proportion of heifers older than 15 months that are not pregnant | 0 1 2 3 4 5 6 7 8 9 10 |
|  | % of open heifers > 16 months | Average proportion of heifers older than 16 months that are not pregnant | 0 1 2 3 4 5 6 7 8 9 10 |
|  | % of open heifers > 17 months | Average proportion of heifers older than 17 months that are not pregnant | 0 1 2 3 4 5 6 7 8 9 10 |
|  | % of heifers <2 standard deviations from 400 kg at 400d | Average proportion of heifers with 2 standard deviations below 400 kg at 400 d | 0 1 2 3 4 5 6 7 8 9 10 |
|  | % of heifers < 580 kg at calving | Average proportion of heifers calving with less than 580 kg | 0 1 2 3 4 5 6 7 8 9 10 |
|  | Heifer efficiency, % | Average proportion of heifers that calved below or at 24 months of age divided by the total number of heifers that were born during that period | 0 1 2 3 4 5 6 7 8 9 10 |
|  | OTHER PARAMETERS USEFUL FOR YOU | Other parameters that the consultant uses and not included in the survey | Text |


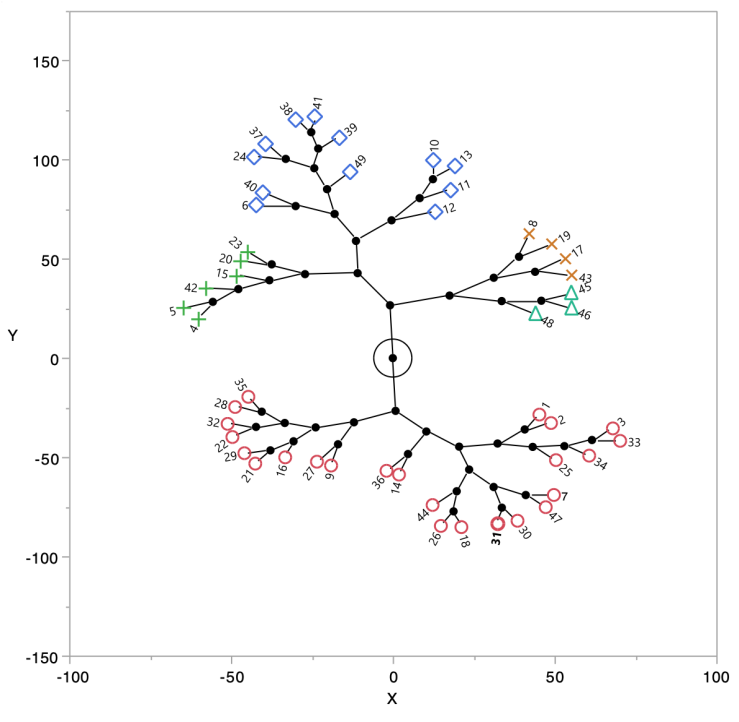


Supplementary Figure 1. Constellation plot showing the results of a hierarchical analysis of the 49 surveys answered of *Section 2. General data of the farm.* The numeration corresponds to each consultant that answered the survey. Cluster 1, 2, 3, 4 and 5 are represented in red circles, green crosses, blue squares, red crosses and green triangles, respectively.

Supplementary Table 2. Answer pattern for the 5 clusters obtained from the 43 parameters evaluated in the *Section 2. General data* *of the farm* after hierarchical clustering analysis. It is detailed in brackets the number of surveys answered belonging to each cluster. Parameters considered as highly important, moderately important, low important and irrelevant are showed in green, blue, orange and red, respectively.

| General Data of the Farm | Cluster 1 (24) | Cluster 2 (6) | Cluster 3 (12) | Cluster 4 (4) | Cluster 5 (3) |
| --- | --- | --- | --- | --- | --- |
| Average number of lactations, n |  |  |  |  |  |
| Total number of cows, n |  |  |  |  |  |
| Number of milking cows, n |  |  |  |  |  |
| Number of 1^st^ lact cows, n |  |  |  |  |  |
| 1^st^ lact cows, % |  |  |  |  |  |
| Dry cows, % |  |  |  |  |  |
| Number of dry cows, n |  |  |  |  |  |
| Monthly milk yield, kg |  |  |  |  |  |
| Daily milk yield, kg |  |  |  |  |  |
| Lactating cows daily milk yield, kg |  |  |  |  |  |
| All cows daily milk yield, kg |  |  |  |  |  |
| Total pregnant cows, n |  |  |  |  |  |
| Pregnant cows, % |  |  |  |  |  |
| Average DIM, DIM |  |  |  |  |  |
| Average days dry, d |  |  |  |  |  |
| 305 day yield, kg |  |  |  |  |  |
| Culling rate, % |  |  |  |  |  |
| Failure to conceive culling rate, % |  |  |  |  |  |
| "Do not breed" cows, % |  |  |  |  |  |
| Cows culled for reproductive reason, % |  |  |  |  |  |
| Cows culled for lameness reason, % |  |  |  |  |  |
| Cows culled for mastitis reason, % |  |  |  |  |  |
| Cows culled for accident reason, % |  |  |  |  |  |
| Average SCC, SCC/mL |  |  |  |  |  |
| Clinical mastitis, % |  |  |  |  |  |
| Lameness, % |  |  |  |  |  |
| Peak milk 1^st^ lact cows, kg |  |  |  |  |  |
| Peak milk 2^nd^ lact cows, kg |  |  |  |  |  |
| Peak milk 3^rd^ lact cows, kg |  |  |  |  |  |
| Peak milk >3^rd^ lact cows, kg |  |  |  |  |  |
| Peak milk 1^st^ lact cows, DIM |  |  |  |  |  |
| Peak milk 2^nd^ lact cows, DIM |  |  |  |  |  |
| Peak milk 3^nd^ lact cows, DIM |  |  |  |  |  |
| Peak milk >3^rd^ lact cows, DIM |  |  |  |  |  |
| Replacement, % |  |  |  |  |  |
| Average lact of culled cows, n |  |  |  |  |  |
| Number of cows culled, n |  |  |  |  |  |
| DIM culled cows, n |  |  |  |  |  |
| Herd status for brucellosis, yes/no |  |  |  |  |  |
| Herd status for neosporosis, yes/no |  |  |  |  |  |
| Herd status for BVDV, yes/no |  |  |  |  |  |
| Herd status for IBR-IPV, yes/no |  |  |  |  |  |
| Herd status for FMD, yes/no |  |  |  |  |  |

DIM, Days in milk; lact, lactation; SCC, Somatic Cell Count; BVDV, Bovine Viral Diarrhea Virus; IBR-IPV, Infectious Bovine Rhinotracheitis - Infectious Pustular Vulvovaginitis; FMD, Foot and Mouth Disease.

|  |  |  |
| --- | --- | --- |


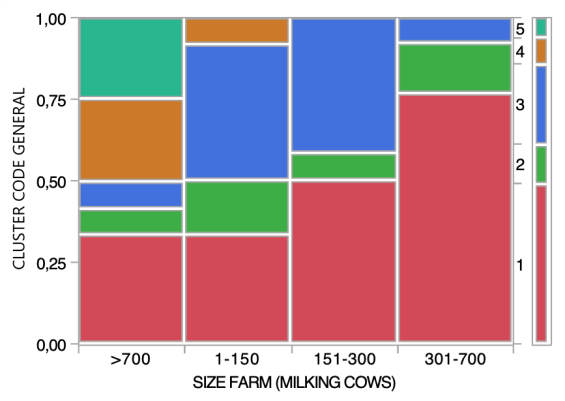


Supplementary Figure 2. Distribution of consultants among the different clusters in section 2, considering the size of the farms advised by the consultants. Cluster 1, 2, 3, 4 and 5 are represented in red, dark green, blue, orange and soft green color, respectively.


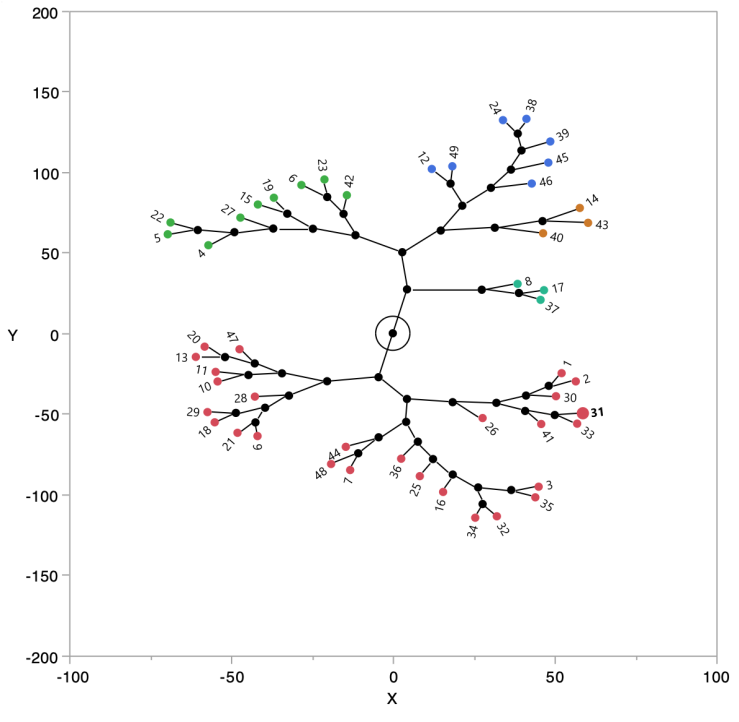


Supplementary Figure 3. Constellation plot showing the results of a hierarchical analysis of the 49 surveys answered of *Section 3. Cow reproduction.* The numeration corresponds to each consultant that answered the survey. Cluster 1, 2, 3, 4 and 5 are represented in red, dark green, blue, brown and soft green color, respectively.

Table S3. Answer pattern for the 5 clusters obtained from the 49 parameters evaluated in the *Section 3. Cow Reproduction* after hierarchical clustering analysis. It is detailed in brackets the number of surveys answered belonging to each cluster. Parameters considered as highly important, moderately important, low important and irrelevant are showed in green, blue, orange and red, respectively.

| Cows’ Reproduction Parameter | Cluster 1 (27) | Cluster 2 (9) | Cluster 3 (7) | Cluster 4 (3) | Cluster 5 (3) |
| --- | --- | --- | --- | --- | --- |
| Voluntary waiting period, d |  |  |  |  |  |
| Percent conceiving of served, % |  |  |  |  |  |
| Overall pregnancy rate, % |  |  |  |  |  |
| First service CR, % |  |  |  |  |  |
| Days open, n |  |  |  |  |  |
| Days to culling, n |  |  |  |  |  |
| Non- return rate, % |  |  |  |  |  |
| CR, % |  |  |  |  |  |
| Ovarian cysts, % |  |  |  |  |  |
| Anovulatory cows, % |  |  |  |  |  |
| 21d Pregnancy rate, % |  |  |  |  |  |
| Services per pregnancy, n |  |  |  |  |  |
| Pregnancy loss, % |  |  |  |  |  |
| Early pregnancy loss (1-42 days), % |  |  |  |  |  |
| Pregnancy loss (1-90days), % |  |  |  |  |  |
| Abortion >90 days, % |  |  |  |  |  |
| CR synchronized cows, % |  |  |  |  |  |
| CR of the sire, % |  |  |  |  |  |
| CR of inseminators, % |  |  |  |  |  |
| Days at pregnancy diagnosis, n |  |  |  |  |  |
| CR first service in 1^st^ lact cows, % |  |  |  |  |  |
| CR first service in 2^nd^ lact cows, % |  |  |  |  |  |
| CR first service in 3^rd^ lact cows, % |  |  |  |  |  |
| CR first service in >3^rd^ lact cows, % |  |  |  |  |  |
| CR first service in multiparous cows, % |  |  |  |  |  |
| CR 1^st^ lact cows, % |  |  |  |  |  |
| CR 2^nd^ lact cows, % |  |  |  |  |  |
| CR 3^rd^ lact cows, % |  |  |  |  |  |
| CR >3^rd^ lact cows, % |  |  |  |  |  |
| CR multiparous cows, % |  |  |  |  |  |
| Submission rate first 3 weeks, % |  |  |  |  |  |
| Submission rate, % |  |  |  |  |  |
| Calving to first service interval, d |  |  |  |  |  |
| Interval heat to heat, d |  |  |  |  |  |
| Interval service to service, d |  |  |  |  |  |
| Heat detection rate, % |  |  |  |  |  |
| 2–17 d. return to service/heat, % |  |  |  |  |  |
| 18–24 d. return to service/heat, % |  |  |  |  |  |
| 25–35 d. return to service/heat, % |  |  |  |  |  |
| 36–48 d. return to service/heat, % |  |  |  |  |  |
| >49 d. return to service/heat, % |  |  |  |  |  |
| 100-Day In-calf rate, % |  |  |  |  |  |
| Cows served <90 DIM, % |  |  |  |  |  |
| Herd calving to conception interval, d |  |  |  |  |  |
| Calving interval, d |  |  |  |  |  |
| Cows not pregnant >200 DIM, % |  |  |  |  |  |
| Cows not pregnant >150 DIM, % |  |  |  |  |  |
| Calvings per month, n |  |  |  |  |  |
| 1^st^ lact cows calved, % |  |  |  |  |  |

CR, Conception rate; DIM, Days in milk; lact, lactation


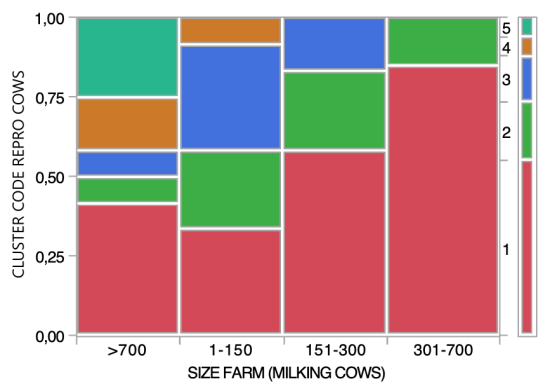


Supplementary Figure 4. Distribution of consultants among the different clusters in section 3, considering the size of the farms advised by the consultants. Clusters 1, 2, 3, 4 and 5 are represented in red, dark green, blue, orange and soft green color, respectively.


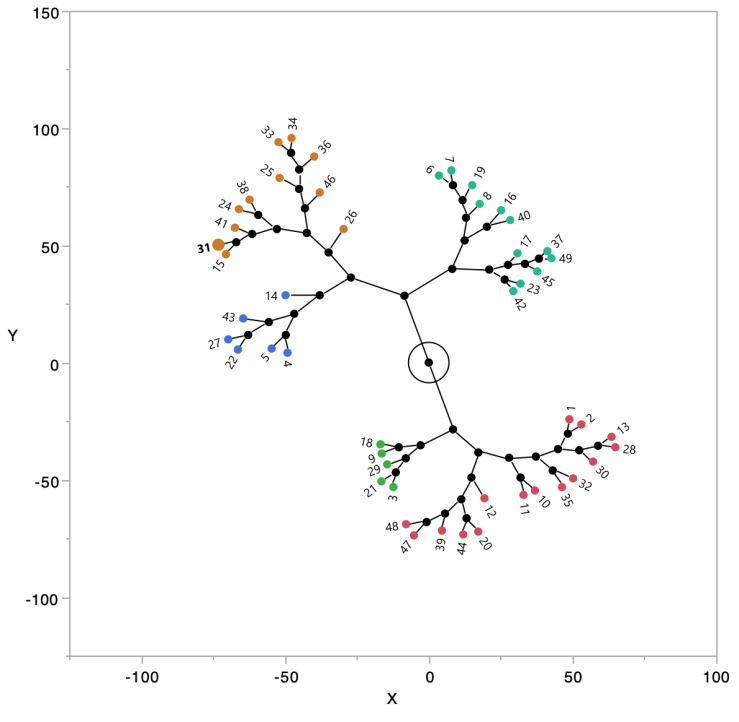


Supplementary Figure 5. Constellation plot showing the results of a hierarchical analysis of the 49 surveys answered of *Section 4. Postpartum and metabolic diseases.* The numeration corresponds to each consultant that answered the survey. Cluster 1, 2, 3, 4 and 5 are represented in red, dark green, blue, brown and soft green color, respectively.

Supplementary Table 4. Answer pattern for the 5 clusters obtained from the 36 parameters evaluated in the *Section 4. Postpartum and metabolic diseases* after hierarchical clustering analysis. It is detailed in brackets the number of surveys answered belonging to each cluster. Parameters considered as highly important, moderately important, low important and irrelevant are showed in green, blue, orange and red, respectively.

| Postpartum and metabolic disease parameter | Cluster 1 (15) | Cluster 2 (5) | Cluster 3 (6) | Cluster 4 (11) | Cluster 5 (12) |
| --- | --- | --- | --- | --- | --- |
| Metritis, % |  |  |  |  |  |
| Retained placenta, % |  |  |  |  |  |
| CK, % |  |  |  |  |  |
| CK and SCK, % |  |  |  |  |  |
| Hypocalcaemia, % |  |  |  |  |  |
| Stillbirth, % |  |  |  |  |  |
| Twins, % |  |  |  |  |  |
| Dystocia, % |  |  |  |  |  |
| Metritis 1^st^ lact, % |  |  |  |  |  |
| Retained placenta 1^st^ lact cows, % |  |  |  |  |  |
| CK 1^st^ lact cows, % |  |  |  |  |  |
| CK and SCK 1^st^ lact cows, % |  |  |  |  |  |
| Hypocalcaemia 1^st^ lact cows, % |  |  |  |  |  |
| Stillbirth 1^st^ lact cows, % |  |  |  |  |  |
| Twins 1^st^ lact cows, % |  |  |  |  |  |
| Dystocia 1^st^ lact cows, % |  |  |  |  |  |
| Metritis multiparous cows, % |  |  |  |  |  |
| Retained placenta multiparous cows, % |  |  |  |  |  |
| CK multiparous cows, % |  |  |  |  |  |
| CK and SCK multiparous cows, % |  |  |  |  |  |
| Hypocalcaemia multiparous cows, % |  |  |  |  |  |
| Stillbirth multiparous cows, % |  |  |  |  |  |
| Twins multiparous cows, % |  |  |  |  |  |
| Dystocia multiparous cows, % |  |  |  |  |  |
| Inc uterine Inv > 30DIM, % |  |  |  |  |  |
| Inc uterune Inv > 30DIM 1^st^ lact cows, % |  |  |  |  |  |
| Inc uterine Inv > 30DIM multiparous cows, % |  |  |  |  |  |
| Pyometra, % |  |  |  |  |  |
| Pyometra 1^st^ lact cows, % |  |  |  |  |  |
| Pyometra multiparous cows, % |  |  |  |  |  |
| Perineal injury, % |  |  |  |  |  |
| Perineal injury 1^st^ lact cows, % |  |  |  |  |  |
| Perineal injury multiparous cows, % |  |  |  |  |  |
| Abomasal pathology, % |  |  |  |  |  |
| Abomasal pathology 1^st^ lact cows, % |  |  |  |  |  |
| Abomasal pathology multiparous cows, % |  |  |  |  |  |

CK, Clinical ketosis; DIM, Days in milk; lact, lactation; Inc uterine Inv, Incorrect uterine involution; SCK, Subclinical ketosis.

A B


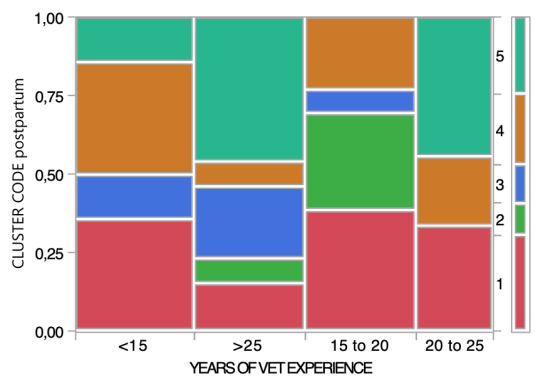

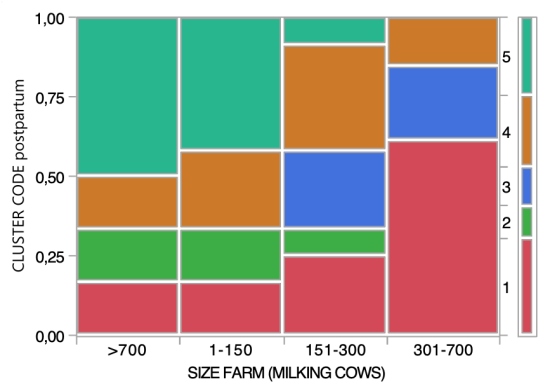


Supplementary Figure 6. Distribution of consultants among the different clusters in section 4, considering the years of experience (A) and the size of the farms they advise (B). Cluster 1, 2, 3, 4 and 5 are represented in red, dark green, blue, orange and soft green color, respectively.


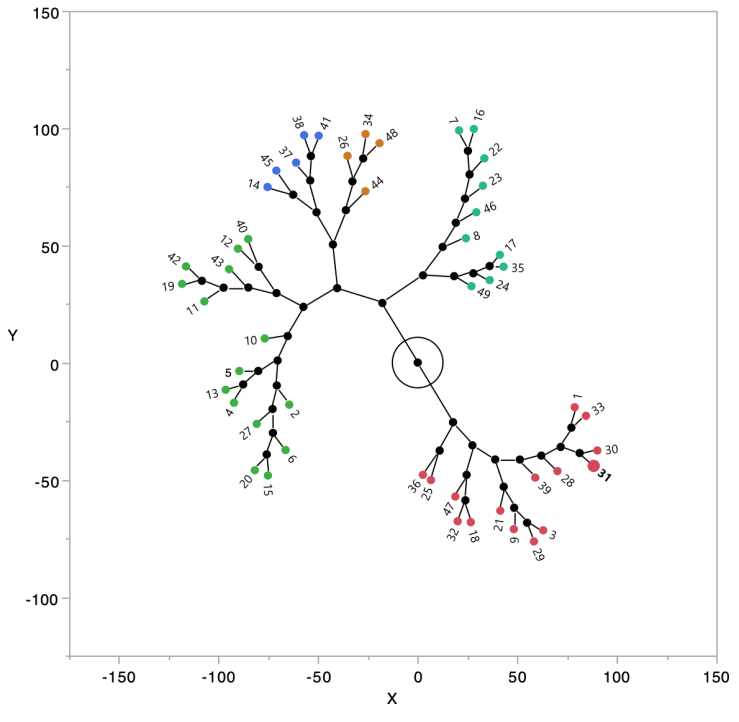


Supplementary Figure 7. Constellation plot showing the results of a hierarchical analysis of the 49 surveys answered of *Section 5. Heifer reproduction.* The numeration corresponds to each consultant that answered the survey. Cluster 1, 2, 3, 4 and 5 are represented in red, dark green, blue, brown and soft green color, respectively.

Supplementary Table 5. Answer pattern for the 5 clusters obtained from the 50 parameters evaluated in the *Section 5. Heifer reproduction* after hierarchical clustering analysis. It is detailed in brackets the number of surveys answered belonging to each cluster. Parameters considered as highly important, moderately important, low important and irrelevant are showed in green, blue, orange and red, respectively.

| Heifers’ Reproduction Parameter | Cluster 1 (16) | Cluster 2 (14) | Cluster 3 (5) | Cluster 4 (4) | Cluster 5 (10) |
| --- | --- | --- | --- | --- | --- |
| CR, % |  |  |  |  |  |
| First service CR, % |  |  |  |  |  |
| Interval heat to heat, d |  |  |  |  |  |
| Interval service to service, d |  |  |  |  |  |
| Heat detection rate, % |  |  |  |  |  |
| Ovarian cysts, % |  |  |  |  |  |
| Anovulatory heifers, % |  |  |  |  |  |
| 21d Pregnancy rate, % |  |  |  |  |  |
| Service pregnant heifer, n |  |  |  |  |  |
| Culling rate heifers, % |  |  |  |  |  |
| Heifers culled for reproductive reason, % |  |  |  |  |  |
| "do not breed" heifers, % |  |  |  |  |  |
| Age at first service, d |  |  |  |  |  |
| Age at first calving, d |  |  |  |  |  |
| Heifers calving <24 months old, n |  |  |  |  |  |
| Heifers calving <23 months old, n |  |  |  |  |  |
| Heifers calving <22 months old, n |  |  |  |  |  |
| Heifers calving <21 months old, n |  |  |  |  |  |
| CR synchronized heifers, % |  |  |  |  |  |
| CR of the sire, % |  |  |  |  |  |
| CR of inseminators, % |  |  |  |  |  |
| Days at pregnancy diagnosis, d |  |  |  |  |  |
| Number of heifers, n |  |  |  |  |  |
| Heifers/Cows, % |  |  |  |  |  |
| Heifers <14 months old, % |  |  |  |  |  |
| Heifers >14 months old, % |  |  |  |  |  |
| Heifers <13 months old, % |  |  |  |  |  |
| Heifers >13 months old, % |  |  |  |  |  |
| Heifers <12 months old, % |  |  |  |  |  |
| Heifers >12 months old, % |  |  |  |  |  |
| Heifers <11 months old, % |  |  |  |  |  |
| Heifers >11 months old, % |  |  |  |  |  |
| Heifers >14 months old not serviced, % |  |  |  |  |  |
| Heifers >13 months old not serviced, % |  |  |  |  |  |
| Heifers >12 months old not serviced, % |  |  |  |  |  |
| Heifers >11 months old not serviced, % |  |  |  |  |  |
| Heifers pregnant, % |  |  |  |  |  |
| Pregnancy loss, % |  |  |  |  |  |
| Early pregnancy loss (1-42 days), % |  |  |  |  |  |
| Pregnancy loss (1-90days), % |  |  |  |  |  |
| Abortion >90 days, % |  |  |  |  |  |
| Open heifers > 12 months, % |  |  |  |  |  |
| Open heifers > 13 months, % |  |  |  |  |  |
| Open heifers > 14 months, % |  |  |  |  |  |
| Open heifers > 15 months, % |  |  |  |  |  |
| Open heifers > 16 months, % |  |  |  |  |  |
| Open heifers > 17 months, % |  |  |  |  |  |
| Heifers <2 SD from 400 kg at 400 d, % |  |  |  |  |  |
| Heifers < 580 kg at calving, % |  |  |  |  |  |
| Heifer efficiency, % |  |  |  |  |  |

CR, Conception rate; SD: Standard deviation.
